# Biochemical and genetic studies define the functions of methylthiotransferases in methanogenic and methanotrophic archaea

**DOI:** 10.3389/fmicb.2023.1304671

**Published:** 2023-11-23

**Authors:** Kaleb Boswinkle, Thuc-Anh Dinh, Kylie D. Allen

**Affiliations:** Department of Biochemistry, Virginia Tech, Blacksburg, VA, United States

**Keywords:** methanogens, ANME archaea, methylthiotransferases, radical SAM enzymes, tRNA modifications

## Abstract

Methylthiotransferases (MTTases) are radical *S*-adenosylmethionine (SAM) enzymes that catalyze the addition of a methylthio (-SCH_3_) group to an unreactive carbon center. These enzymes are responsible for the production of 2-methylthioadenosine (ms^2^A) derivatives found at position A37 of select tRNAs in all domains of life. Additionally, some bacteria contain the RimO MTTase that catalyzes the methylthiolation of the S12 ribosomal protein. Although the functions of MTTases in bacteria and eukaryotes have been established via detailed genetic and biochemical studies, MTTases from the archaeal domain of life are understudied and the substrate specificity determinants of MTTases remain unclear. Here, we report the *in vitro* enzymatic activities of an MTTase (C4B56_05415) from a thermophilic *Ca.* Methanophagales anaerobic methanotroph (ANME) as well as the MTTase from a hyperthermophilic methanogen – MJ0867 from *Methanocaldococcus jannaschii*. Both enzymes catalyze the methylthiolation of *N^6^*-threonylcarbamoyladenosine (t^6^A) and *N^6^-*hydroxynorvalylcarbamoyladenosine (hn^6^A) residues to produce 2-methylthio-*N^6^*-threonylcarbamoyladenosine (ms^2^t^6^A) and 2-methylthio-*N^6^-*hydroxynorvalylcarbamoyladenosine (ms^2^hn^6^A), respectively. To further assess the function of archaeal MTTases, we analyzed select tRNA modifications in a model methanogen – *Methanosarcina acetivorans* – and generated a deletion of the MTTase-encoding gene (MA1153). We found that *M. acetivorans* produces ms^2^hn^6^A in exponential phase of growth, but does not produce ms^2^t^6^A in detectable amounts. Upon deletion of MA1153, the ms^2^A modification was absent, thus confirming the function of MtaB-family MTTases in generating ms^2^hn^6^A modified nucleosides in select tRNAs.

## Introduction

**Figure 1:**
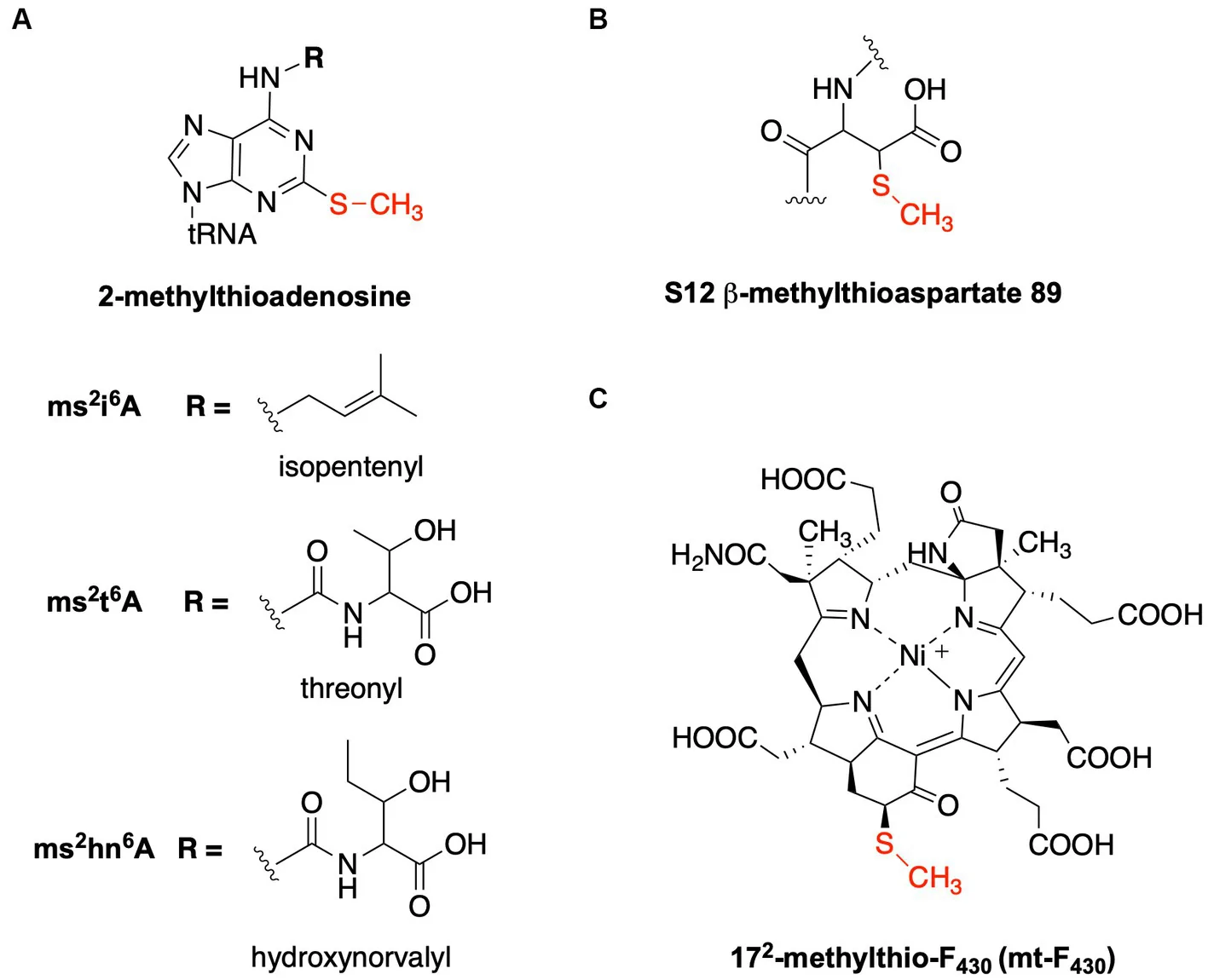
Structures of methylthiolated biomolecules. **(A)** 2-methylthioadenosine found at position 37 of specific tRNAs. The methylthiolated versions of three different modified nucleosides are well described: 2-methylthio-*N^6^*-isopentenyladenosine (ms^2^i^6^A), 2-methylthio-*N^6^*-threonylcarbamoyladenosine (ms^2^t^6^A), and 2-methylthio-*N^6^-*hydroxynorvalylcarbamoyladenosine (ms^2^hn^6^A). **(B)** β-methythioaspartate is found at position 89 of the S12 ribosomal protein in many bacteria. **(C)** 17^2^-methylthio-F_430_ is present in ANME-1 and functions with methyl-coenzyme M reductase.

Sulfur is a versatile element found in a range of biomolecules including the amino acids methionine and cysteine, and cofactors such as iron–sulfur (Fe-S) clusters, biotin, lipoic acid, thiamine, coenzyme A, and molybdopterin. Additionally, various sulfur-containing modifications exist on tRNA including derivatives where the keto oxygen on the base is replaced with sulfur as well as 2-methylthio (ms^2^) derivatives of adenosine. The latter methylthiolated nucleosides include 2-methylthio-*N^6^*-isopentenyladenosine (ms^2^i^6^A), 2-methylthio-*N^6^*-threonylcarbamoyladenosine (ms^2^t^6^A), and 2-methylthio-*N^6^-*hydroxynorvalylcarbamoyladenosine (ms^2^hn^6^A) (Figure 1), as well as a handful of other less common methylthiolated derivatives (Cavuzic and Liu, 2017; Zheng et al., 2017; Boccaletto and Baginski, 2021). The ms^2^i^6^A modification is widely distributed in bacteria and in mitochondrial tRNAs of some eukaryotes, while ms^2^t^6^A is found in some bacteria as well as eukaryotes and archaea (Jackman and Alfonzo, 2013; Wei et al., 2015; Landgraf et al., 2016). Some organisms, such as *Bacillus subtilis*, produce both ms^2^i^6^A and ms^2^t^6^A, where the former is present in tRNA^Phe(GAA)^ and tRNA^Tyr(QUA)^ and the latter is found in tRNA^Lys(UUU)^ (Vold et al., 1982; Boccaletto and Baginski, 2021). Although seemingly less common, hn^6^A and ms^2^hn^6^A have been identified in several bacteria and archaea (Reddy et al., 1992; McCloskey et al., 2001; Noon et al., 2003; Yu et al., 2019; Boccaletto and Baginski, 2021). The i^6^A, t^6^A, and hn^6^A modifications, with or without the associated ms^2^ derivatives, are found at position A37 of UNN-decoding tRNAs (i^6^A and ms^2^i^6^A) and ANN-decoding tRNAs (t^6^A, ms^2^t^6^A, hn^6^A, and ms^2^hn^6^A) (Schweizer et al., 2017; Yu et al., 2019; Su et al., 2022). Position 37 is 3′-adjacent to the anticodon and these modifications function to stabilize relatively weak A:U and U:A base pairs in order to enhance translational fidelity (Zheng et al., 2017; Su et al., 2022).

Methanogens are a diverse group of archaea with ancient evolutionary origins (Battistuzzi et al., 2004; Adam et al., 2017). They are found in a wide range of anaerobic environments including marine and freshwater ecosystems, anoxic soils, anaerobic sewage digesters, and as important components of animal microbiomes (Lyu et al., 2018; Moissl-Eichinger et al., 2018; Borrel et al., 2020). As their sole source of energy, methanogens carry out a form of anaerobic respiration known as methanogenesis, which reduces simple oxidized carbon compounds to generate methane as an end product. Another group of archaea related to methanogens carry out the anaerobic oxidation of methane (AOM) and are known as anaerobic methanotrophs (ANME) (Timmers et al., 2017). Consistent with their anaerobic lifestyles and ancient evolutionary origins, methanogens (and the related ANME) contain an abundance of Fe-S cluster proteins (Major et al., 2004; Imlay, 2006; Liu et al., 2010, 2012; Johnson et al., 2021). Additionally, these organisms contain two unique sulfur-containing biomolecules, coenzyme M and coenzyme B, which are cofactors of methyl-coenzyme M reductase – the key methane-forming enzyme of methanogenesis and the initial methane activation enzyme of AOM (Thauer, 2019). The key catalytic component of MCR is coenzyme F_430_, the nickel tetrahydrocorphin prosthetic group. Interestingly, some ANME – the ANME-1 clade [*Ca.* Methanophagales (Adam et al., 2017)] – utilize a modified form of F_430_ that contains a methylthio group at the 17^2^ position (Figure 1) (Mayr et al., 2008; Shima et al., 2012). The impact of this modification on MCR catalysis is unknown, but it is appealing to propose that the modification could play a role in tuning the potential catalytic bias in ANME-1 MCR to perform methane oxidation as opposed to methane formation. However, it is important to note that other clades of ANME appear to utilize the canonical, unmodified F_430_ (Kaneko et al., 2014; Gendron and Allen, 2022).

**Figure 2:**
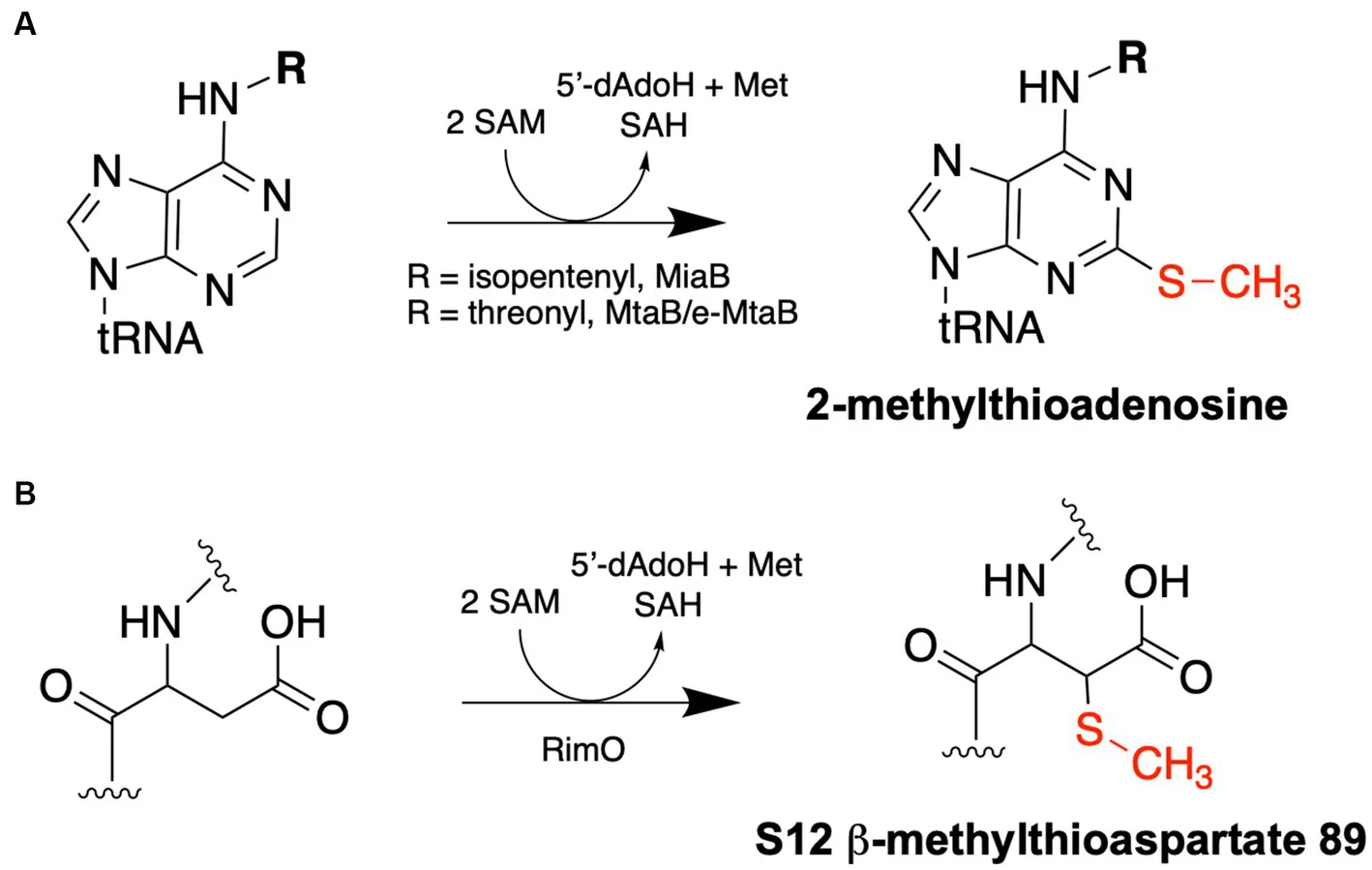
Methylthiotransferase reaction schemes. **(A)** tRNA MTTases – MiaB and MtaB/e-MtaB – catalyze the methylthiolation of A37 residues of specific tRNAs. **(B)** RimO catalyzes the methylthiolation of the β carbon of Asp89 of the S12 ribosomal protein. Each reaction requires two molecules of SAM: one is used as a methyl group donor to synthesize the methylthio group and produce SAH as a product, while the other is used for radical SAM chemistry to append the methylthio group to the respective substrate and produce 5′-deoxyadenosine (5′-dAdoH) and methionine as products.

The methylthiolation reactions for ubiquitous tRNA modifications described above, as well as for the related methylthiolation of the S12 ribosomal protein (Figure 1) occurring in select bacteria, are catalyzed by methylthiotransferase (MTTase) enzymes belonging to the radical *S*-adenosylmethionine (SAM) superfamily (Figure 2). Radical SAM enzymes use SAM and a four-iron four-sulfur ([4Fe-4S]_RS_) cluster – most commonly bound by three cysteines in a CX_3_CX_2_C motif – to generate a highly reactive 5′-deoxyadenosyl radical (5′-dAdo•) that facilitates complex radical chemistry to catalyze a wide range of reactions in all domains of life (Sofia et al., 2001; Holliday et al., 2018; Oberg et al., 2022). In addition to the [4Fe-4S]_RS_ cluster, MTTases contain an “auxiliary” cluster ([4Fe-4S]_aux_) that is involved in sulfur incorporation (Forouhar et al., 2013; Landgraf et al., 2013; Zhang et al., 2020). There are four major clades of MTTases based upon phylogenetic analysis of the MTTase family (Anton et al., 2010; Arragain et al., 2010). RimO is exclusively bacterial and is the only known MTTase that does not act upon a tRNA substrate. Instead, it is responsible for the methylthiolation of D88 of ribosomal protein S12 (Anton et al., 2008). MiaB, found in bacteria and eukaryotic organelles, utilizes i^6^A-containing tRNA as a substrate to generate ms^2^i^6^A. The final two clades are responsible for methylthiolation of t^6^A-containing tRNA in bacteria (MtaB) and archaea/eukaryotes (e-MtaB). It remains unclear which MTTases are responsible for the production of ms^2^hn^6^A; however, based on the structural similarity of the substrate and the distribution among organisms that produce ms^2^hn^6^A, it is proposed that members of MtaB and e-MtaB clades can perform this reaction (Anton et al., 2008).

Here, we report the biochemical characterization of the MTTase homolog encoded in the genome of the G60 ANME-1 archaeon (Krukenberg et al., 2018) as well as the *in vitro* activity of the MTTase from the hyperthermophilic methanogen *Methanocaldococcus jannaschii* (Bult et al., 1996). Additionally, we generated a deletion of the putative MTTase in *Methanosarcina acetivorans* to confirm the function of an archaeal MTTase *in vivo*. Although we had originally proposed that the ANME-1 MTTase may be responsible for the methylthiolation of F_430_ to produce 17^2^-methylthio-F_430_ (mt-F_430_, Figure 1), we have not observed evidence for this activity and instead confirm that the MTTases studied here are responsible for the methylthiolation of select tRNAs to produce ms^2^t^6^A and/or ms^2^hn^6^A.

## Materials and methods

### Chemicals

Ampicillin, kanamycin, isopropyl-β-D-thiogalactoside (IPTG), and dithiothreitol (DTT) were from Gold Biotechnology. *S*-adenosylmethionine (SAM) hydrochloride, FMN, FeCl_3_, cysteine, sodium dithionite, as well as trace elements and vitamins for methanogen media were from Millipore-Sigma. NADPH was from Cayman Chemical Company. DNA oligonucleotides and gBlocks were obtained from Integrated DNA Technologies (IDT). All other standard reagents and supplies were from Genesee Scientific or Research Products International unless stated otherwise.

### Strains and growth procedures

Transformed strains of *E. coli* DH5α (Zymo research) and *E. coli*-CodonPlus (DE3)-RIL (Agilent Technologies) were grown in LB (Miller) medium with the appropriate antibiotic(s). A *Bacillus subtilis* strain lacking the MtaB-encoding gene (*B. subtilis* 168 *ΔyqeV::kan*, BKK25430) (Koo et al., 2017) was obtained from the Bacillus Genetic Stock Center and grown in LB medium with 7.5 μg/mL kanamycin. *Methanococcus maripaludis* S2 Mm902 (Walters et al., 2011; Tunckanat et al., 2022) was obtained from the laboratory of Dr. John Leigh (University of Washington) and grown in McCas medium (Moore and Leigh, 2005) with H_2_/CO_2_ (80/20, 40 psi). *M. maripaludis* S2 Mm902 was constructed as reported in Walters et al. (2011), except the ORF1 was inserted into the *upt* gene (MMP0680) in the S2 wild-type strain instead of *M. maripaludis* S2 Mm900 (Moore and Leigh, 2005). *Methanosarcina acetivorans* WWM60 was obtained from Dr. Biswarup Mukhopadhyay (Virginia Tech, originally from W. W. Metcalf, University of Illinois at Urbana-Champaign) and was grown on high-salt medium (Sowers et al., 1993) in methanol (100 mM) or trimethylamine (50 mM). For the analysis of the ms^2^hn^6^A modification, *M. acetivorans* (as well as the ΔMA1153 strain) was grown in methanol and harvested in log phase at an OD_600_ of 0.7. For anaerobic growth of methanogens (Wolfe, 2011), 20 mL Balch tubes (Chemglass), 125 mL serum bottles (Wheaton), or 1 L bottles with a Balch-type closure (Chemglass) were used.

### General molecular biology procedures

For expression of putative MTTases in *E. coli*, the respective genes were cloned into pET15b. For expression of putative MTTases in *M. maripaludis* S2 Mm902, the respective genes were cloned into pJAR50CT constructs [a derivative of pMEV1 (Sarmiento et al., 2011)]. For generation of the MA1153 deletion in *M. acetivorans* WWM60, the pDN201 Cas9 construct was used followed by recombination into pAMG40 (described further below) (Nayak and Metcalf, 2017; Nayak and Metcalf, 2018). All constructs were created with Gibson assembly (Gibson et al., 2009) using the HiFi Master Mix (New England Biolabs) and the protocol recommended by the supplier. More detailed molecular biology procedures, along with the sequences of gBlocks (Supplementary Table S1) and primers (Supplementary Table S2), as applicable, can be found in the supplementary material.

### G60 ANME-1 MTTase expression and purification

The G60 ANME-1 MTTase was expressed with an N-terminal hexahistidine tag from pET15b in *E. coli*-CodonPlus (*DE3*)-RIL. Transformed cells were grown routinely in 3 L flasks containing 1.5 L LB supplemented with 100 μg/mL ampicillin at 37°C with shaking at 200 RPM. At an OD_600_ of ~0.4, cysteine and FeCl_3_ were added to final concentrations of 25 μM each. Then, at an OD of ~0.7, expression was induced with IPTG (100 μM). Cells were grown for an additional four hours at 37°C and then harvested by centrifugation. The cell pellet was stored at −20°C for future purification.

Purification of the G60 ANME-1 MTTase was carried out inside an anaerobic chamber (Coy Laboratory Products) with an atmosphere of 97% N_2_ and 3% H_2_. Buffer A consisted of 50 mM HEPES, 500 mM KCl, 10 mM imidazole, 5 mM DTT, pH 8.0 and Buffer B was the same except with 500 mM imidazole. The cell pellet (14 g) was resuspended in 40 mL of anaerobic buffer A and sonicated on ice. A Misonex sonicator equipped with a microtip was employed with the duty cycle set at 50 (%/1 s) and the power at 4 (microtip limit). Cells were sonicated 15 times for 2 min each with a one-minute rest between each sonication round. Phenylmethylsulfonyl fluoride (1 mM) was added after the first round of sonication. Homogenized cells were transferred to a sealed anaerobic centrifuge tube and centrifuged at 27,000 x *g* (30 min). The supernatant was filtered by passing it through a syringe connected to a 0.45 μm filter and then applied to a gravity flow column containing 2 × 6 cm of Ni Sepharose 6 Fast Flow resin (Cytiva). The column was washed with 15 mL buffer A, then eluted in a stepwise gradient of increasing buffer B consisting of 6 mL fractions of 5% buffer B, 10% buffer B, 20% buffer B, 50% buffer B, and finally 100% buffer B. The G60 ANME-1 MTTase eluted in the 20% B and 50% B fractions, which were combined and concentrated with a 30 kD Amicon Ultra centrifugal filter unit (Millipore-Sigma). The protein was finally exchanged into 50 mM HEPES, 500 mM KCl, 5 mM DTT, pH 8.0 using a PD-10 desalt column (Cytiva). Purifications were assessed by SDS-PAGE and protein concentration was determined using a Bradford assay (Bradford, 1976). The amount of iron bound to purified protein was determined using a well described ferrozine assay (Fish, 1988). For storage, the protein was aliquoted into 1 mL anaerobic cryovials containing O-ring seals (Olympus) then flash frozen in liquid N_2_ and stored at −80°C.

### *Methanococcus maripaludis* culture, transformation, and *Mj*MTTase purification

*Methanococcus maripaludis* S2 Mm902 was transformed with pJAR50 MTTase constructs (BSM_21210 or MJ0867) using polyethylene glycol-mediated transformation procedures (Sarmiento et al., 2011) with 1 μg of purified plasmid and a 5 mL overnight culture. The transformations were plated on McCas agar medium in an anaerobic chamber with an atmosphere of 77% N_2_/20% CO_2_/3% H_2_ followed by incubation at 37°C in a sealed pressurized vessel containing a paper towel soaked in 10% Na_2_S and an atmosphere of H_2_/CO_2_ (80/20) (30 psi). Colonies appeared after ~3 days. After verification of successful transformation by PCR, the strains were cultured in 1 L anaerobic bottles (Chemglass) containing 300 mL McCas medium supplemented with 0.125 mg/mL puromycin with 40 psi H_2_/CO_2_ (80/20) at 37°C with gentle shaking until OD_600_ ~ 1.0. Cells were harvested anaerobically via centrifugation in sealed bottles and the pellet was used immediately for protein purification.

The *Mj*MTTase (MJ0687) was expressed from pKB604 (derived from pJAR50CT, see methods in Supplementary material for more details), under the control of P_hmva_, with a C-terminal twin strep tag. A typical purification was from 1.2 L of culture (4 × 300 mL). Cells (~1.5 g) were harvested anaerobically in sealed centrifuge bottles and the pellet was resuspended in 5 mL Strep buffer W (100 mM Tris, 150 mM NaCl, pH 8.0). The cells were lysed by sonication on ice followed by centrifugation at 27,000 *x g* for 45 min. The supernatant was filtered and applied to a gravity flow column containing 2 × 6 cm of Strep-Tactin Sepharose resin (IBA Lifesciences). The column was washed 5 times, each with 2 mL of Strep buffer W. The protein was then eluted in 6 fractions, each with 1 mL of Strep buffer W containing 2.5 mM desthiobiotin. *Mj*MTTase eluted in fractions 2 and 3, which were then combined and concentrated with a 30 kD Amicon Ultra centrifugal filter unit. The concentrated protein was then exchanged into 50 mM HEPES, 300 mM KCl, 5 mM DTT, pH 8.0 using a PD-10 desalt column (Cytiva). The final protein sample was stored as aliquots of 1 mL in anaerobic cryovials, flash frozen in liquid N_2_, and stored at -80 °C.

### Electron paramagnetic resonance (EPR) spectroscopy

Samples (300 μL) were prepared in the anaerobic chamber and contained 108 μM G60 ANME-1 MTTase in 50 mM HEPES, 300 mM KCl, pH 8.0. When applicable, sodium dithionite and SAM were added at concentrations of 4 mM and 1 mM, respectively. The reactions were incubated at room temperature for 10 min and then transferred to 4 mm EPR tubes (Norrell), frozen in cold isopentane (~77 K), and stored in liquid N_2_. Low temperature X-band EPR spectra were obtained on a Bruker ER073 EMX Spectrophotometer with an EMX High Sensitivity Probehead and a liquid helium variable temperature system (ER4112HV). Spectra were recorded under the following conditions: 9.376 GHz, 10 G modulation amplitude, 3,400 G center field, 1,000 G sweep width, 1 mW power, 0.3 s time constant, 10 K, 3 × 1 min scans.

### *In vitro* enzyme assays with coenzyme F_430_

Reactions with purified F_430_ were carried out to assess whether the G60 ANME-1 MTTase catalyzed the synthesis of mt-F_430_. Coenzyme F_430_ was purified from *M. maripaludis* cells using the following procedure. Cells (~1 g wet weight) were resuspended in 3 mL H_2_O and lysed by sonication. Formic acid was added to a final concentration of 1% followed by centrifugation at 4,400 x *g*. The supernatant was neutralized with NaOH, and diluted 2x with 50 mM Tris, pH 7.5. The resulting sample was filtered and then applied to a gravity flow column with Q Sepharose Fast Flow resin (2 × 10 cm) equilibrated with 50 mM Tris, pH 7.5. After washing with 10 mL of 50 mM Tris, pH 7.5, the F_430_ was eluted with 6 mL of 20 mM formic acid. After removal of formic acid via evaporation under vacuum at 30°C (down to ~500 μL), the concentration of F_430_ was determined by using an extinction coefficient of 23.0 mM^−1^ cm^−1^ at 430 nm (Pfaltz et al., 1982, 1987). These preparations contain some sarcinapterin (methanopterin with a glutamate residue) in addition to F_430_. Enzyme reactions with the resulting F_430_ and purified MTTase were carried out in an anaerobic chamber in a reaction volume of 500 μL in 50 mM HEPES, 300 mM KCl, 5 mM DTT, pH 8.0. Reactions contained the following components: G60 ANME-1 MTTase (50 μM), coenzyme F_430_ (~75 μM), SAM (1 mM), sodium sulfide (500 μM), and a reducing agent – either sodium dithionite (2 mM), titanium citrate (2 mM), or NAPDH (2 mM) and FMN (50 μM). The latter biologically relevant reducing system has recently been reported to support radical SAM enzyme catalysis *in vitro* (Eastman et al., 2023). It was an especially desirable option for this reaction since the normal reducing agent – sodium dithionite – also reduces the F_430_. In one set of reactions, *M. acetivorans* cell extract was also supplied in addition to these components. To prepare the methanogen cell extract, cells (1.2 g) were resuspended in 3 mL of reaction buffer and sonicated on ice (duty cycle set at 50 (%/1 s) and the power at 4, four rounds of 30 s sonication with 1 min rest between each round). Then, 100 μL of this extract was added to the reaction containing the additional components listed above (three reactions with cell extract were set up with each containing a different reducing agent described above). Reactions were incubated at 50°C for three hours or 18 h at 37°C followed by the addition of 1 volume acetonitrile. After removing the precipitated protein by centrifugation, the supernatant was concentrated to ~100 μL by drying at 30°C under vacuum.

For LC–MS analysis of F_430_ reactions, a Waters Acquity UPLC with a TQD mass spectrometer equipped with an Acquity Premier BEH C18 (2.1 × 75 mm, 1.7 μm) column was employed. Solvent A was 0.1% (v/v) formic acid in water and solvent B was 100% methanol. The flow rate was 0.35 mL/min and the LC program consisted of 2 min at 95% A followed by a 10 min linear gradient to 50% B, then a 3 min gradient to 95% B. The injection volume was typically 2 μL. The source temperature was 150°C, the desolvation temperature was 500°C, the desolvation gas flow was 800 L/h, and the cone gas flow was 50 L/h. The mass spectrometer was operated in positive mode and scanned for *m/z* values ranging from 400 to 1,200.

### tRNA purification and digestion

Protocols were adapted from published procedures (Yu et al., 2019; Avcilar-Kucukgoze et al., 2020). All solutions for tRNA preparations were treated with diethyl pyrocarbonate (DEPC) to 0.1% with stirring overnight followed by autoclaving. A typical tRNA purification from *M. acetivorans* cells used a ~ 1 g cell pellet while a typical tRNA purification from *B. subtilis* cells used a ~ 5 g cell pellet. Cell pellets were washed with 0.9% NaCl (6 mL/g of cells) then resuspended in 50 mM sodium acetate, 10 mM magnesium acetate, pH 5 (4 mL/g of cells). *M. acetivorans* cells, but not *B. subtilis* cells, were sonicated at this stage. For sonication, a Misonex sonicator equipped with a microtip was employed with the duty cycle set at 50 (%/1 s) and the power at 4 (microtip limit). Cells were sonicated on ice three times for 30 s each with a one-minute rest between. To this suspension was added an equal amount saturated phenol in 0.1 M citrate, pH 4.3 (Millipore-Sigma). The emulsion was incubated with shaking (200 RPM) for 15 min at room temperature, followed by centrifugation at 5,000 × *g* (15 min). The upper aqueous phase was transferred to a new tube and 5 mL chloroform was added, followed by centrifugation again to remove the remaining phenol. The aqueous phase was removed, adjusted to 0.2 M NaCl, followed by addition of 1 volume isopropanol. This was incubated at −20°C for 30 min followed by centrifugation at 5,000 × *g* (15 min). The pellet was washed with 70% ethanol then resuspended in 50 mM sodium acetate, 10 mM MgCl_2_, and 150 mM NaCl pH 6.5 (2 mL/g of cells). LiCl was added to a final concentration of 2 M and the sample was incubated on ice for one hour, followed by centrifugation at 10,000 × *g* for 15 min. The supernatant containing RNA was transferred to a new tube and 1 volume isopropanol was added followed by incubation at −20°C for 30 min and centrifugation at 10,000 × *g*. The pellet was washed with 70% ethanol, then resuspended in molecular grade water. To further purify tRNAs from this mixture, the solution was subjected to anion exchange chromatography using the NucleoBond RNA/DNA 400 kit (Macherey-Nagel) according to the manufacturer’s instructions. The resulting tRNA was resuspended in 100 μL water, then stored at −80°C.

To analyze modification profiles of extracts, tRNAs were digested and dephosphorylated to the respective nucleosides. Purified tRNA (100 μg) in a volume of 40 μL was incubated at 100°C for three minutes and then cooled in an ice-water bath. The pH was adjusted with 4 μL of 100 mM ammonium acetate, pH 5.3, followed by the addition of 10 U nuclease P1 (New England Biolabs) and incubation at 37°C overnight (at least 12 hours). Then, 5 μL 100 mM ammonium bicarbonate was added followed by 0.01 U of *Crotalus adamanteus* phosphodiesterase I (Millipore-Sigma) and incubation at 42°C for two hours. Then, 1 U alkaline phosphatase (calf intestinal; Promega) was added and this was incubated for an additional two hours. Finally, this solution was concentrated to 35 μL by drying at 30°C under vacuum followed by LC–MS analysis.

LC–MS analysis of nucleosides was carried out using a Waters Acquity UPLC with a TQD mass spectrometer equipped with an Acquity Premier BEH C18 (2.1 × 75 mm, 1.7 μm) column. Solvent A was 0.1% (v/v) formic acid in water and solvent B was 100% methanol. The flow rate was 0.35 mL/min and the LC program consisted of 2 min at 98% A followed by a 13 min linear gradient to 75% B, then a 15 min gradient to 100% B and 3 min at 100% B. The injection volume was typically 5 μL. The source temperature was 150°C, the desolations temperature was 500°C, the desolvation gas flow was 800 L/h, and the cone gas flow was 50 L/h. The mass spectrometer was operated in positive mode and scanned for *m/z* values ranging from 100 to 800. The main ions detected by mass spectrophotometry are the molecular ion and the base fragment ion. These two *m/z* values are well characterized for dozens of nucleosides. These masses in combination with elution times are used to identify specific nucleosides.

### *In vitro* enzyme assays with tRNA

Reactions (150 μL) were carried out in anaerobic conditions in 50 mM HEPES, 500 mM KCl, pH 8 with purified tRNA from either the *B. subtilis* Δ*mtaB* strain (BKK25430) (100 μg tRNA per reaction) or from the *M. acetivorans* ΔMA1153 strain (50 μg tRNA per reaction). The G60 ANME-1 MTTase reactions contained 40 μM protein, while the *Mj*MTTase reactions contained 10 μM protein. The additional reaction components consisted of: 500 μM sodium sulfide, 1 mM SAM, and a reducing agent (3 mM sodium dithionite or 2 mM NADPH and 50 μM FMN). The reactions were incubated at 50°C for 3 h, after which protein was precipitated with addition of 150 μL (1 vol) of acetonitrile. After centrifugation, the supernatant was transferred to a new tube and concentrated to ~40 μL by drying under vacuum at 45°C. RNA was isolated via ethanol precipitation, then digested to nucleosides as described above.

### Generation of a MA1153 deletion in *Methanosarcina acetivorans*

Cas9-mediated genome editing tools developed by Nayak and Metcalf (2017, 2018) were used to delete the putative MTTase encoding gene (MA1153) in *M. acetivorans* WWM60. The single-guide RNA (sgRNA) construct and the homology directed repair (HDR) template were obtained as gBlocks from IDT (Supplementary Table S1) and designed with appropriate overlaps for Gibson assembly. The sgRNA construct was designed with the following elements: the *mtaCB1* promoter, two sgRNAs separated by a 30-bp linker, and the *mtaCB1* terminator. This was assembled into pDN201 (Nayak and Metcalf, 2017, 2018) using the AscI restriction site. After confirmation of successful insertion of the sgRNA component, the construct was digested with PmeI followed by assembly with the HDR template. The construct was designed to leave 60 bp at each end of the gene to maintain potential promoter and terminator elements for surrounding genes. The sequence was confirmed by whole-plasmid sequencing (Plasmidsaurus - Eugene, OR). The sequence-verified plasmid was then recombined with pAMG40 (Guss et al., 2008) followed by restriction digest confirmation and transformation into *M. acetivorans* WWM60 (Guss et al., 2008) via liposome-mediated transformation procedures (Metcalf et al., 1997; Buan et al., 2011) with 10 mL of a late exponential phase culture and 2 μg of plasmid DNA. Transformants were plated on high-salt agar medium in an anaerobic chamber and plates were incubated at 37°C in a sealed vessel containing N_2_/CO_2_ (80/20) (20 psi) and a paper towel soaked in a 10% solution of Na_2_S. Colonies appeared after 12 days. The desired MA1153 deletion strain was confirmed by PCR (Supplementary Figure S1) using primers flanking the deletion site (Supplementary Table S2) as well as Sanger sequencing of the PCR product.

## Results

### Identification and initial characterization of an ANME-1 MTTase

In this work, we originally set out to identify the putative MTTase required for 17^2^-methylthio-F_430_ biosynthesis (mt-F_430_, Figure 1). Analysis of available metagenome data revealed a single likely MTTase encoded in ANME-1 genomes (Supplementary Table S3), of the ANME-2 genomes, only ANME-2a have a significant MTTase homolog (Supplementary Table S3). Since mt-F_430_ is established to be present in ANME-1 organisms, while being absent in most ANME-2-rich environments (Mayr et al., 2008; Shima et al., 2012; Allen et al., 2014; Kaneko et al., 2014), we hypothesized that the MTTase present in ANME-1 genomes would be responsible for the biosynthesis of mt-F_430_. The structure of mt-F_430_ was originally determined by isolating the cofactor from ANME-rich microbial mats collected at methane seeps in the Black Sea (Mayr et al., 2008). Later, mt-F_430_ was identified in the crystal structure of the ANME-1 MCR purified from the Black Sea mat biomass (Shima et al., 2012). Thus, we initially chose to investigate BSM_21210, the MTTase encoded in the Black Sea mat ANME-1 genome (Meyerdierks et al., 2010). BSM_21210 shares 30% identity and 48% similarity to MtaB from *B. subtilis* and 38% identity and 55% similarity to e-MtaB (MJ0867) from *Methanocaldococcus jannashii* (see sequence alignment in Supplementary Figure S2). With the goal of obtaining purified protein for enzyme activity studies, the BSM_21210 gene was assembled into pET15b and heterologously expressed in *E. coli*. Multiple expression and purification conditions were attempted, but although the enzyme exhibited high level expression, it was present in the insoluble fraction, and we were unable to obtain purified protein suitable for *in vitro* studies. We also attempted to express and purify the protein in a model methanogen, *M. maripaludis*. Unfortunately, both N-terminally and C-terminally hexahistidine-tagged versions of the BSM MTTase, under the control of the constitutive promoter P*hmva*, expressed at very low levels in *M. maripaludis* and we could not recover enough protein for *in vitro* studies.

**Figure 3:**
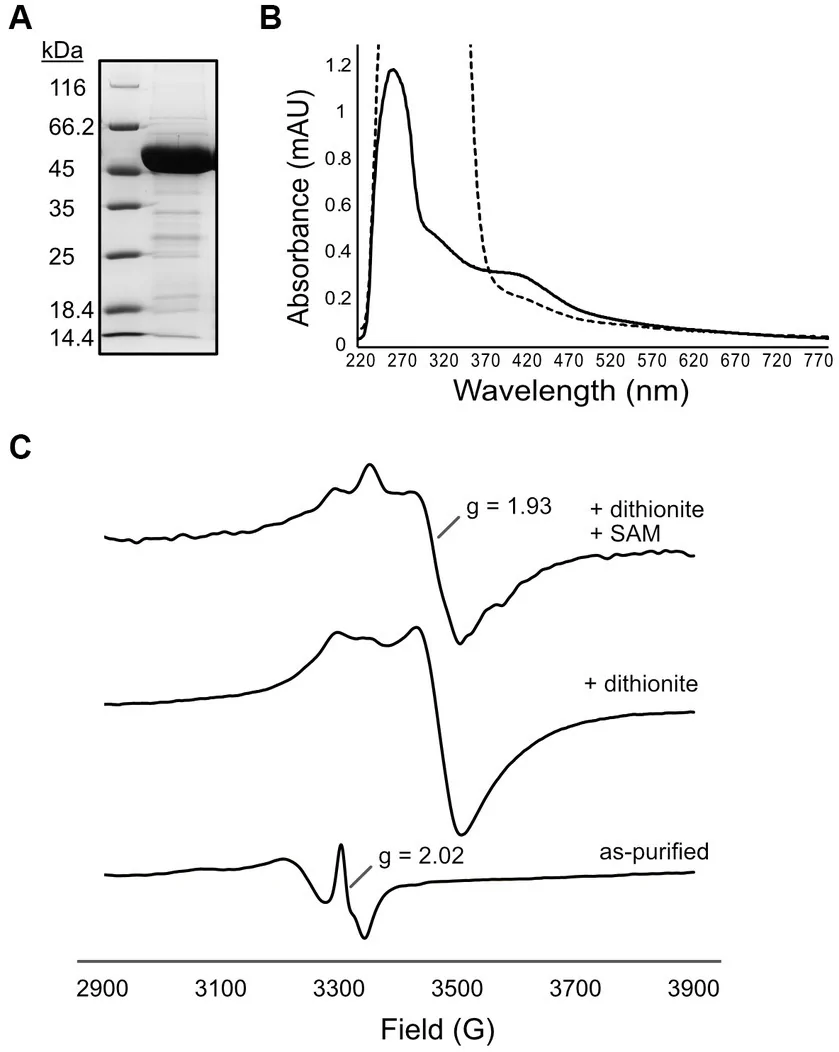
Purification and characterization of [4Fe-4S] cluster(s) of G60 ANME-1 MTTase. **(A)** SDS-PAGE gel stained with Coomassie showing purified his-tagged G60 ANME-1 MTTase which migrates consistent with the expected molecular weight of 50 kDa (including N-terminal tag). **(B)** UV–Vis spectrum of as-purified protein (solid line) compared to dithionite-reduced (dashed line). **(C)** Electron paramagnetic resonance spectroscopy of as-purified protein (bottom spectrum) compared to in the presence of dithionite (middle spectrum) and dithionite + SAM (top spectrum).

Since we encountered difficulties with BSM_21210, we next focused our studies on a homologous MTTase from a thermophilic ANME-1 that would hopefully be more amenable to biochemical investigation. Krukenburg et al. cultivated and characterized an ANME-1a thermophile from Guaymas basin hydrothermal sediment that grows at 60°C (“G60”) (Krukenberg et al., 2018). Small molecule extracts from these cultures were previously demonstrated to contain mt-F_430_ (Allen et al., 2014). Analysis of the genome revealed a likely MTTase – C4B56_05415 (PXF52554.1) – that shares 63% identity and 78% similarity to BSM_21210. Additionally, this is the only homolog of any sulfur-inserting radical SAM enzyme in the G60 ANME-1 genome (Supplementary Table S4). Thus, we assembled the gene into pET15b with an N-terminal hexahistidine-tag for expression in *E. coli*-CodonPlus (DE3)-RIL. The G60 ANME-1 MTTase was highly expressed in a soluble form and we typically obtained about 5 mg of purified protein per gram (wet weight) of *E. coli* cells (Figure 3A). Additionally, the protein had a prominent brown color, a hallmark of iron–sulfur cluster containing enzymes, and the UV–Vis spectrum showed a broad absorbance band at ~420 nm (Figure 3B, solid line), characteristic of the expected [4Fe-4S]^2+^ clusters. Upon reduction with sodium dithionite, the 420 nm peak was bleached (Figure 3B, dashed line), indicating reduction to the [4Fe-4S]^1+^ state. Further characterization of the Fe-S cluster bound by the G60 ANME-1 MTTase using electron paramagnetic resonance (EPR) spectroscopy showed that the as-purified enzyme contains a signal indicative of a [3Fe-4S]^1+^ cluster(s) (Figure 3C, bottom spectrum) (Doan et al., 1994; Liu and Graslund, 2000). Upon reduction with sodium dithionite, an EPR signal at g ~ 1.93 is observed, which corresponds to reduced [4Fe-4S]^1+^ cluster(s), and the addition of SAM results in subtle lineshape changes consistent with the interaction of SAM with the radical SAM cluster (Figure 3C, middle and top spectra, respectively) (Broderick et al., 2014). A ferrozine assay for iron determination showed that the purified G60 ANME-1 MTTase contained 5.8 ± 0.4 mol Fe per mol protein. Although not ideal for the expected two [4Fe-4S] clusters, this value suggested that at least a portion of the purified protein was sufficiently loaded with the correct cofactors and, together with the spectroscopic data reported above, we were confident moving forward with enzymatic activity assays.

### *In vitro* methylthiolation activity of the G60 ANME-1 MTTase

**Figure 4:**
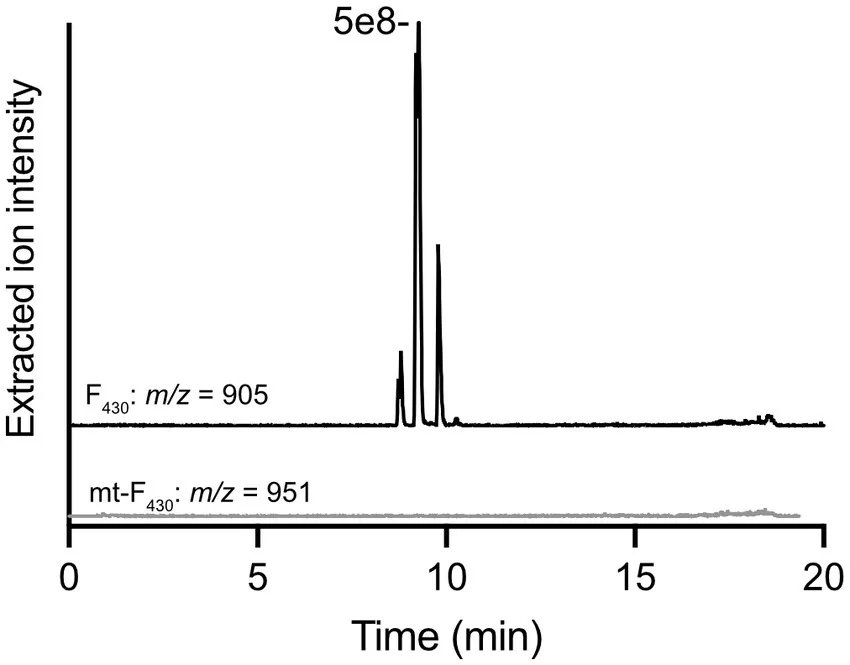
*In vitro* enzyme assay with G60 ANME-1 MTTase and F_430_. LC–MS analysis of a representative reaction with purified F_430_ as a potential substrate. Extracted ion chromatograms are shown for F_430_ (*m/z* 905) – multiple peaks are observed due to isomerization occurring during the incubation at 50°C for the enzyme reaction – and the potential product, mt-F_430_ (*m/z* 951). Multiple reaction conditions were tested and are discussed in the materials and methods.

With the purified G60 ANME-1 MTTase in hand, *in vitro* enzyme assays with purified F_430_ obtained from methanogen cells were performed. We tried several different reaction conditions (various reducing systems and the addition of methanogen cell lysates, see materials and methods for complete description); however, the production of mt-F_430_ by this putative MTTase was never observed (see representative LC–MS results in Figure 4). Based on our experience with the unmodified coenzyme, the limit of detection for mt-F_430_ in our LC–MS experiment is expected to be ~1 pmol – this would correspond to 0.1 μM product produced in the enzyme assay containing ~75 μM F_430_ and 50 μM protein (1.25 mg in 500 μL reaction). Thus, our negative results suggest that the MTTase homolog encoded in ANME-1 genomes is not responsible for the synthesis of mt-F_430_.

Since the ANME-1 MTTase did not appear to catalyze the methylthiolation of F_430_, we next considered that the enzyme instead acted upon a tRNA substrate to catalyze the production of 2-methylthiolated adenosine (ms^2^A) residues at position A37 of select tRNA(s) (Figure 1). As mentioned above, available ANME-1 metagenomes contain a single MTTase homolog and, although the ms^2^A tRNA modification has not specifically been analyzed in ANME, the modification is widespread in all domains of life. The MTTases present in archaea are members of the “e-MtaB” clade and are responsible for the methylthiolation of t^6^A to produce ms^2^t^6^A (Anton et al., 2010; Arragain et al., 2010). It was important to confirm this activity so that we could more confidently rule out a function in F_430_ modification.

**Figure 5:**
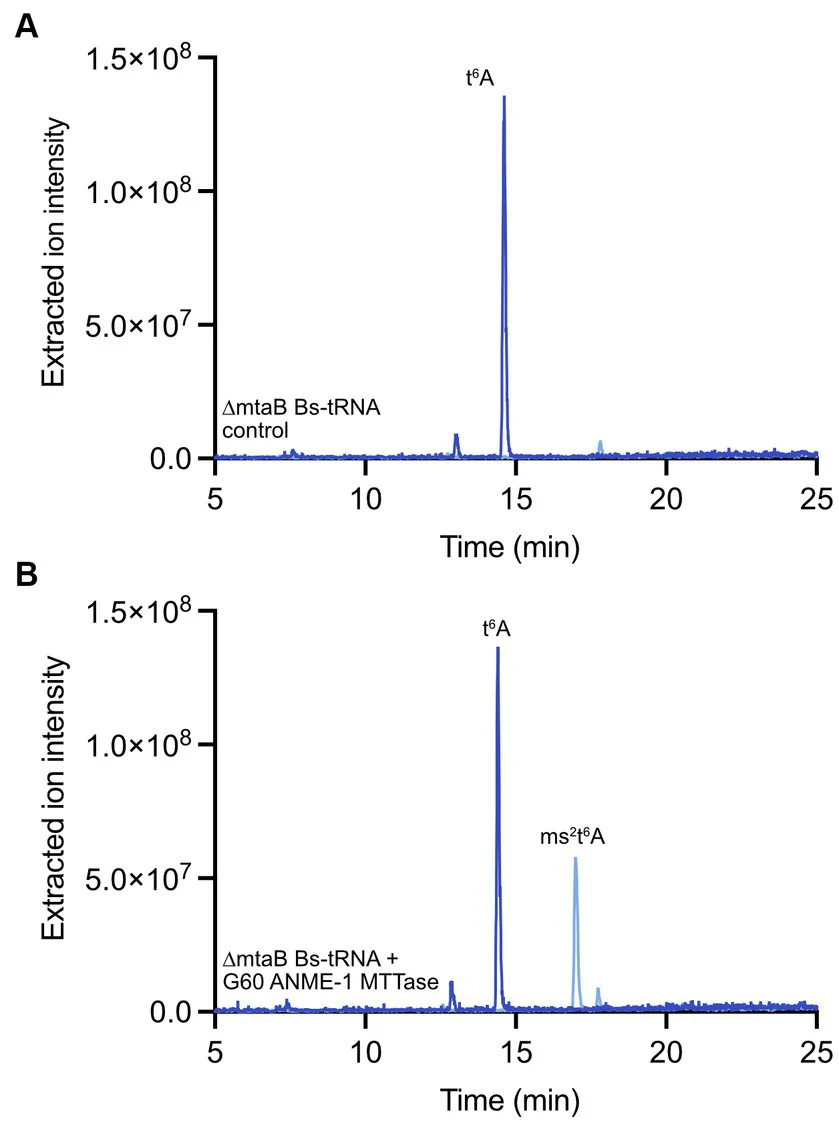
*In vitro* enzyme assay with G60 ANME-1 MTTase and tRNA. LC–MS analysis of a representative reaction with bulk tRNA from a *B. subtilis* ΔmtaB strain. Overlaid extracted ion chromatograms are shown for t^6^A (*m/z* 413, dark blue) and ms^2^t^6^A (*m/z* 459, light blue) for **(A)** the control reaction lacking the enzyme compared to **(B)** the full reaction containing enzyme. See materials and methods for detailed reaction conditions and sample processing.

To obtain the putative tRNA substrate for *in vitro* reactions with the G60 ANME-1 MTTase, we obtained a *Bacillus subtilis* strain that lacks the gene encoding MtaB - *B. subtilis* 168 *ΔyqeV::kan* (BKK25430, here we will call this strain ΔmtaB for clarity). In this organism, the ms^2^t^6^A modification is present at position A37 in tRNA^Lys (UUU).^ Thus, deletion of the gene results in tRNA^Lys (UUU)^ with the t^6^A modification, but lacking the ms^2^ modification. When we incubated the purified tRNA from *B. subtilis* ΔmtaB with our purified enzyme in the presence of dithionite and SAM, a clear peak was observed for ms^2^t^6^A that corresponds to the ms^2^t^6^A found in wild-type *B. subtilis*, but is absent in the control reaction lacking enzyme (Figure 5). Thus, this result demonstrates that the G60 ANME-1 MTTase catalyzes the methylthiolation of t^6^A-contining tRNA^Lys (UUU)^. Importantly, it also demonstrates that the enzyme was active in our *in vitro* reaction conditions, allowing us to conclude that the enzyme is likely not responsible for methylthiolation of F_430_.

### *In vivo* confirmation of MTTase function in *Methanosarcina acetivorans*

Although tRNA modification research in archaea is limited, both ms^2^t^6^A as well as ms^2^hn^6^A have been identified in organisms belonging to this domain of life (Reddy et al., 1992; Noon et al., 2003; Yu et al., 2019). Archaeal genomes generally encode only one MTTase, but it is unclear whether the same enzyme is responsible for the production of both methylthiolated nucleosides. MJ0867 – e-MtaB in *M. jannaschii* – was shown to rescue the production of ms^2^t^6^A in a strain of *B. subtilis* lacking MtaB activity, demonstrating that this enzyme can use t^6^A-containing tRNA as a substrate (Anton et al., 2010), which is also consistent with the *in vitro* result reported above with the G60 ANME-1 MTTase. To gain further insights into the function of archaeal MTTases in the synthesis of ms^2^A modified tRNAs, we chose to investigate the *in vivo* function of the MTTase in *M. acetivorans* – a model methanogen with robust genetic tools. The Cas9-mediated genome editing tools developed by Nayak and Metcalf (Nayak and Metcalf, 2017) were used to generate a deletion of the MTTase homolog (MA1153). Transfer RNA from the resulting *M. acetivorans* ΔMA1153 strain was then purified and digested to nucleosides for LC–MS analysis compared to wild-type.

**Figure 6:**
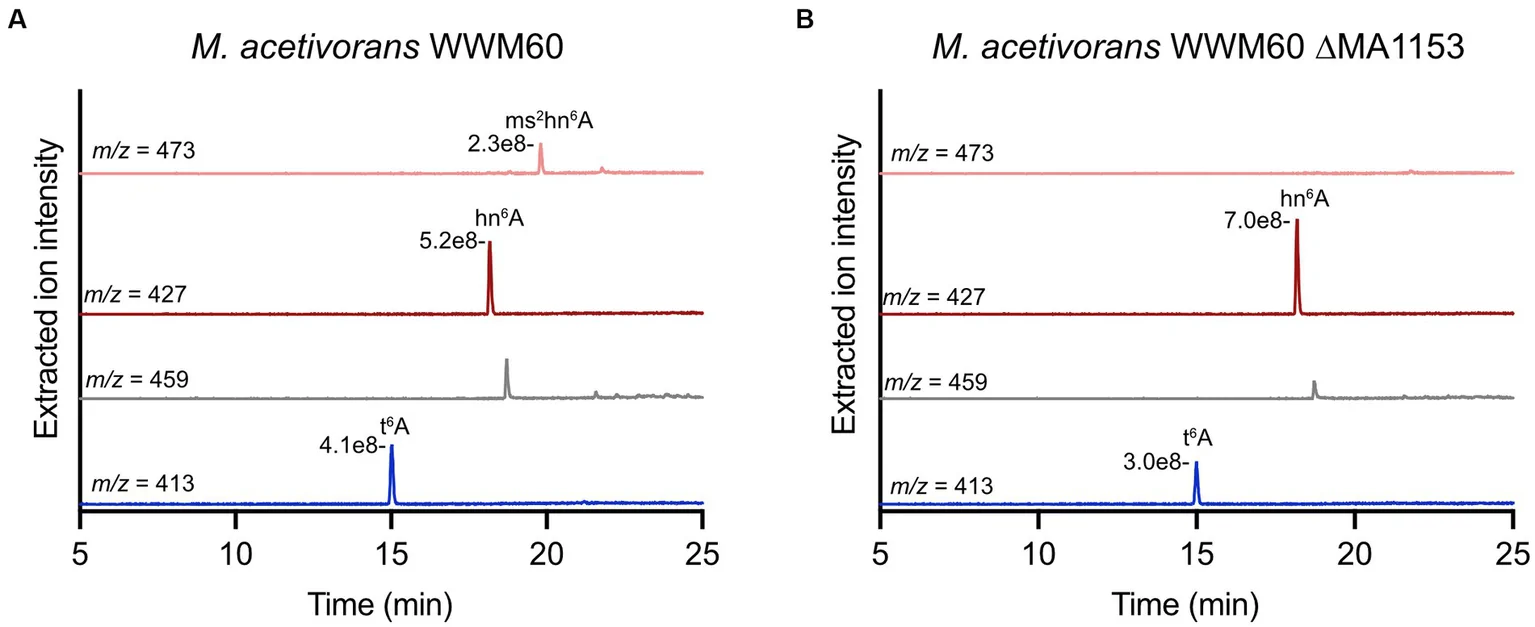
Modified nucleosides in *Methanosarcina acetivorans* WWM60 compared to the ΔMA1153 strain. Extracted ion chromatograms are shown for modified nucleosides of interest in **(A)** *M. acetivorans* WWM60 compared to **(B)** the ΔMA1153 strain. The respective peak intensities are labeled. Both strains were grown in high-salt medium with 100 mM methanol at 37°C to an OD_600_ of 0.7. The peak in the *m/z* 459 chromatogram is not ms^2^t^6^A (see Figure S4).

Analysis of tRNA modifications of interest in *M. acetivorans* WWM60 (wild-type) revealed t^6^A, hn^6^A, and ms^2^hn^6^A, but not ms^2^t^6^A (Figure 6A). It is important to note that hn^6^A has the same mass as m^6^t^6^A, which can make its assignment more challenging. We confirmed the identity of hn^6^A in *M. acetivorans* through high-resolution LC–MS analysis, which revealed an exact mass of *m/z* 427.1575 (expected is 427.1572) as well as, most importantly, the characteristic fragment ions at *m/z* 136.0615 and *m/z* 162.0409 that correspond to the adenine nucleobase without a methyl group and are consistent with the assignment of hn^6^A (Noon et al., 2003; Yu et al., 2019) (Supplementary Figure S3). It is also important to note that the extracted ion chromatogram for the ms^2^t^6^A mass (*m/z* 456) contains a peak (Figure 6); however, it eluted about 0.8 min after the true ms^2^t^6^A and it lacks the characteristic fragment ions. Most notably, the *m/z* 327 ion, corresponding to the modified adenine base fragment ion, is absent (see mass spectra in Supplementary Figure S4). Interestingly, ms^2^hn^6^A was only identified in cells isolated in exponential phase of growth and was not observed in stationary phase cultures. Analysis of the *M. acetivorans* ΔMA1153 strain revealed the absence of ms^2^hn^6^A (Figure 6B), thus supporting the role of this MTTase in generating the ms^2^hn^6^A modification. The *M. acetivorans* ΔMA1153 strain (which still contains the genome-editing plasmid) exhibited moderately slower growth compared to wild-type when grown in standard high salt medium with methanol at 37°C (see growth curve in Supplementary Figure S5).

### *In vitro* activity of G60 ANME-1 MTTase with hn^6^A-containing tRNA

**Figure 7:**
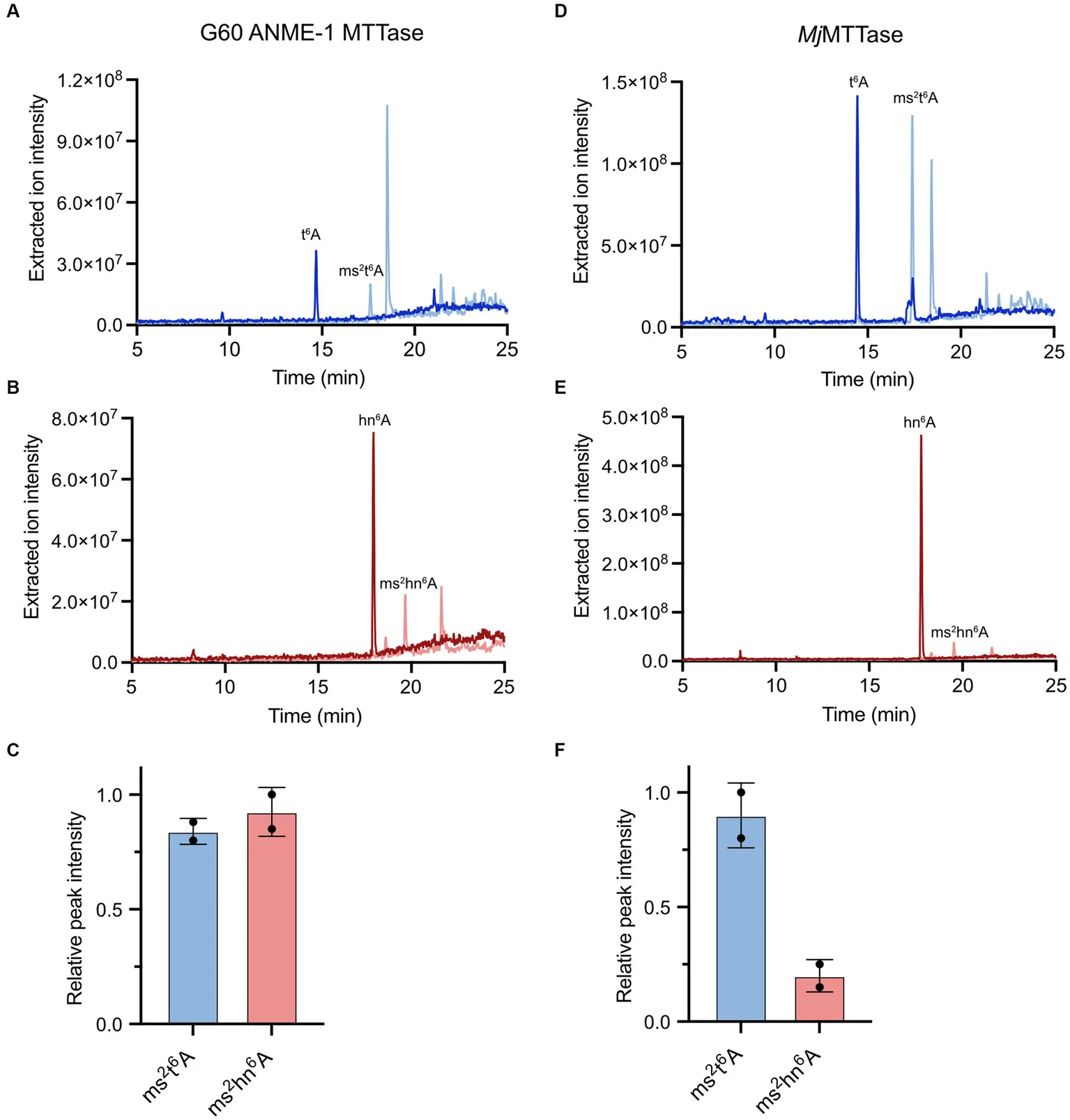
Activities of G60 ANME-1 MTTase and *Mj*MTTase with t^6^A-containing tRNA compared to hn^6^A-containing tRNA. LC–MS analysis of *in vitro* reactions with **(A–C)** G60 ANME-1 MTTase compared to **(D–F)** *Mj*MTTase in the presence of tRNA isolated from *M. acetivorans* ΔMA1153. **(A,D)** Overlaid extracted ion chromatograms for t^6^A and ms^2^t^6^A. **(B,E)** Overlaid extracted ion chromatograms for hn^6^A and ms^2^hn^6^A. **(C,F)** The associated peak intensities are represented in bar graphs for duplicate reactions.

To confirm that the archaeal MTTase could use hn^6^A-containing tRNA as a substrate, we used bulk tRNA from the *M. acetivorans* ΔMA1153 strain in *in vitro* assays with the G60 ANME-1 MTTase. Thus, these reactions contain both t^6^A-containing tRNA as well as hn^6^A-contining tRNA (Figure 6B). Based on the LC–MS analysis of the digested nucleosides, the G60 ANME-1 MTTase catalyzed the production of ms^2^t^6^A as well as ms^2^hn^6^A (Figures 7A-C). Assuming the two nucleosides with highly similar structures have similar ionization efficiencies, the G60 ANME-1 MTTase exhibited similar activities with t^6^A-containing tRNA compared to hn^6^A-containing tRNA as evidenced by the similar peak intensities observed for ms^2^t^6^A compared to ms^2^hn^6^A (Figure 7C).

### *In vitro* enzymatic activity of the *Methanocaldococcus jannaschii* MTTase

To compare the activity of another archaeal MTTase, we chose to investigate MJ0867 from *M. jannaschii* (“*Mj*MTTase”). As mentioned above, the function of this protein in methylthiolation of t^6^A-containing tRNA was previously shown through heterologous expression experiments in *B. subtilis* (Anton et al., 2010). However, recent analysis of tRNA modification in *M. jannaschii* revealed ms^2^t^6^A as a minor component compared to t^6^A, while the amount of ms^2^hn^6^A was similar to hn^6^A (Yu et al., 2019), thus suggesting that MJ0867 prefers the hn^6^A-containing tRNA(s) substrate *in vivo*. This enzyme was not amenable to heterologous expression in *E. coli*; thus, we turned to expression in *M. maripaludis –* a Methanococcales methanogen closely related to *M. jannaschii*. Since there are abundant nickel binding proteins in methanogens, his-tagged protein purifications from these organisms often contain a few major impurities. Thus, *Mj*MTTase was expressed with a C-terminal twin strep tag from pJAR50. The enzyme was successfully expressed and purified (Supplementary Figure S6A) from *M. maripaludis* with a characteristic brown color and a UV–vis spectrum with a broad absorbance band at ~420 nm indicative of a [4Fe-4S] cluster-containing protein (Supplementary Figure S6B). Interestingly, the absorbance spectrum had an intense peak at 260 nm that obscured the 280 nm peak, which we hypothesized was due to the protein binding RNA during the expression and purification process. This will be discussed more later.

*In vitro* enzymatic activity assays revealed that the purified *Mj*MTTase catalyzed the synthesis of ms^2^t^6^A using bulk tRNA from *B. subtilis* ΔmtaB as a substrate (Supplementary Figure S7). Notably, the enzyme demonstrated comparable activities in the presence of the strong chemical reductant, sodium dithionite, as well as NADPH/FMN, a biologically-relevant reducing system recently reported for radical SAM enzyme reduction (Supplementary Figure S7) (Eastman et al., 2023). We also tested the activity of *Mj*MTTase with the tRNA isolated from *M. acetivorans* ΔMA1153. Interestingly, the enzyme exhibited substantially lower activity with hn^6^A-containing tRNA compared to t^6^A-containing tRNA (Figures 7D–F).

To confirm that the methylthiolation activity observed *in vitro* was due to the added tRNA in the reactions and not due to *M. maripaludis* tRNA bound to the purified protein (see 260 nm absorbance in Supplementary Figure S6B), we performed control reactions where all reaction components were added except tRNA. Analysis of digested nucleosides from these control experiments revealed characteristic peaks for the abundant and well-ionizing purine nucleosides – adenosine and guanosine – thus, demonstrating that *Mj*MTTase is purified with bound RNA (Supplementary Figure S8A). However, we did not observe peaks for any of the nucleoside modifications of interest here (Supplementary Figure S8B). Thus, the *in vitro* activity was confirmed to be a result of methylthiolation activity with the added tRNA [either from *B. subtilis* ΔmtaB (Supplementary Figure S7) or *M. acetivorans* ΔMA1153 (Figure 7B)] and not due to the products/substrates being already bound to the purified protein.

## Discussion

Transfer RNAs undergo extensive posttranscriptional modification in all domains of life. Depending on the location of a given modification within the tertiary structure of a tRNA molecule, the modification may have a role in translational fidelity, structural stability, or cellular protection and recognition (Gustilo et al., 2008; Zheng et al., 2017). The methylthio group addition occurs on adenosine residues exclusively at position A37 (Figure 1) in select tRNAs that decode ANN or UNN codons. Modifications at this position stabilize the codon-anticodon interactions, thus enhancing translational fidelity. A37 is typically modified first at the 6 position with an isopentenyl group (i^6^A), a threonyl group (t^6^A), or a hydroxynorvalyl group (hn^6^). Specific tRNAs with these modifications then serve as a substrate for an MTTase enzyme that adds the 2-methylthio group (ms^2^) (Figure 2A). Besides tRNA, the only other known substrate for MTTase family members is the S12 ribosomal protein where the β carbon of Asp88 is methylthiolated by the enzyme RimO (Anton et al., 2008) (Figure 2B).

Structurally, MTTases consist of three domains – an N-terminal MTTase domain, a central radical SAM domain, and a C-terminal TRAM domain that was previously defined as an RNA-binding domain (Anantharaman et al., 2001; Lee et al., 2005). These enzymes bind two [4Fe-4S] clusters – the [4Fe-4S]_RS_ cluster in the radical SAM domain and an auxiliary [4Fe-4S]_aux_ cluster in the N-terminal MTTase domain. MTTases catalyze two distinct half reactions. First, traditional nucleophilic substitution chemistry with SAM generates a methylthio group associated with the auxiliary cluster (Forouhar et al., 2013; Landgraf et al., 2013; Zhang et al., 2020). Then, radical SAM chemistry with a second molecule of SAM produces 5′-dAdo• initiated by an electron from the reduced [4Fe-4S]_RS_ cluster. This radical is proposed to abstract a C2 hydrogen from the A37 residue of the respective tRNA substrate to facilitate installation of the methylthio group (Esakova et al., 2021).

Here, we describe the function of archaeal MTTases through *in vitro* and *in vivo* experiments. We chose to investigate an ANME-1 MTTase to elucidate the substrate(s) of this enzyme and to test our hypothesis that this MTTase could be responsible for the modification of coenzyme F_430_ to generate mt-F_430_ (Figure 1), the coenzyme used by MCR in ANME-1 to initiate AOM (Mayr et al., 2008; Shima et al., 2012). However, *in vitro* enzyme assays with F_430_ did not result in the synthesis of mt-F_430_ by the G60 ANME-1 MTTase, suggesting that the enzyme does not catalyze this reaction. Since this is the only enzyme encoded in ANME-1 genomes with significant homology to any radical SAM sulfur inserting enzyme, this result raises important questions regarding the origin of mt-F_430_ in ANME-1. Non-enzymatic methylthiolation has been described for quinone-containing antibiotics where the required methanethiol is a catabolite of methionine (Li et al., 2013). However, the quinone moiety is essential to activate the molecule for chemical methylthiolation; thus, this chemistry does not seem plausible for F_430_. Indeed, in preliminary experiments with purified F_430_ and methanethiolate, we have not observed evidence for chemical methylthiolation. Another possibility is that F_430_ is not the direct substrate, but instead a precursor in the biosynthetic pathway (Zheng et al., 2016) serves as the substrate for the methylthiolation reaction. However, since unmodified F_430_ as well as mt-F_430_ are detected in ANME-1 cultures (Allen et al., 2014), it seems most likely that F_430_ is the precursor for mt-F_430_. Thus, we currently conclude that yet-to-be discovered enzyme(s) catalyze the methylthiolation of F_430_ and/or that other proteins/cofactors/substrates are required for methylthiolation of F_430_ by the ANME-1 MTTase.

The G60 ANME-1 MTTase as well as *Mj*MTTase catalyzed the methylthiolation of t^6^A-containing tRNA and hn^6^A-containing tRNA to produce ms^2^t^6^A and ms^2^hn^6^A, respectively. Interestingly, *Mj*MTTase seemed to prefer the t^6^A-containing substrate in these experiments. This result was somewhat surprising since the ms^2^hn^6^A modification was shown to be the major ms^2^ species in *M. jannaschii*, whereas ms^2^t^6^A was a more minor component (Yu et al., 2019). However, it is important to note that tRNA modifications have not been mapped in *M. acetivorans*, so it is possible that the ideal substrate for *Mj*MTTase was not present or was not in high enough abundance in our *in vitro* reactions using bulk tRNA from *M. acetivorans*. Ms^2^hn^6^A was mapped to position 37 of tRNA^Met(CAU1)^ in *M. jannaschii*, while ms^2^t^6^A was in too low abundance to be associated with a specific tRNA in this organism (Yu et al., 2019). To our knowledge, ms^2^hn^6^A has not been mapped to specific tRNA(s) in any other organism except *M. jannaschii*.

Our work on these archaeal MTTases supports the previous hypothesis concerning the role of MtaB and e-MtaB clades in the biosynthesis of ms^2^hn^6^A (Anton et al., 2008) and is consistent with previous results that t^6^A and hn^6^A likely share the same biosynthetic pathway (Yu et al., 2019; Swinehart et al., 2020). Although the biosynthetic origin of hydroxynorvaline is unknown, the bacterial enzymes responsible for t^6^A biosynthesis can also perform hn^6^A biosynthesis using provided hydroxynorvaline as a substrate (Swinehart et al., 2020). Thus, the hn^6^A modification is likely incorporated with the same biosynthetic machinery as t^6^A.

An interesting aspect of the MTTase family is understanding what dictates substrate specificity. That is, different tRNAs for MiaB and MtaB/e-MtaB, and a protein substrate for RimO. Although the TRAM domain was originally implicated as playing an important role in RNA-binding, it is now clear that all three MTTase domains are important for substrate binding and recognition. In early studies by Anton et al., chimeric constructs were generated with MiaB and MtaB in *B. subtilis*, and the results suggested that the differential recognition of the i^6^ or t^6^ modification by these enzymes is accomplished by the MTTase domain or the radical SAM domain, and not by the TRAM domain (Anton et al., 2010). More recently, crystal structures of MiaB demonstrated that all three domains of the enzyme are engaged in interactions with the tRNA substrate and specific residues of the enzyme have been identified that provide key interactions to recognize the A36 residue as well as the i^6^ modification of A37 (Esakova et al., 2021). A hydrophobic pocket – consisting of residues from the MTTase domain and the radical SAM domain – was identified to recognize and accommodate the isopentenyl group. It will be important to obtain structures of MtaB and e-MtaB MTTases in the future to further define substrate specificity determinants of MTTases.

Consistent with the structural information described above, previous work demonstrated that MiaB requires the i^6^A modification to be present to catalyze the methylthiolation reaction (Pierrel et al., 2004). However, recently, Koshla et al. reported that the *Streptomyces albidoflavus* J1074 MiaB can methylthiolate unmodified adenosine residues in tRNA to produce ms^2^A (Koshla et al., 2023). Additionally, the presence of ms^2^A has been reported in *E. coli* (Kellner et al., 2014). In our *in vitro* reactions with bulk tRNA from either *B. subtilis* or *M. acetivorans*, we did not detect ms^2^A, suggesting that these archaeal MTTases cannot methylthiolate unmodified adenosine residues. Furthermore, ms^2^A has not been previously reported in archaea and was not observed here in our targeted analysis of *M. acetivorans* tRNA modifications. Other recent work reported the identification of the thiohemiacetal – msms^2^i^6^A – in *E. coli*, a modification shown to be catalyzed by MiaB (Dal Magro et al., 2018). We did not observe evidence for this msms^2^ modification in any of our *in vitro* reactions with the archaeal MTTases studied here. Therefore, the e-MtaB enzymes likely do not catalyze the sequential methylthiolation reaction.

An important observation in this work is that the ms^2^hn^6^A nucleoside was only identified in *M. acetivorans* when cells were harvested in exponential phase of growth and not in stationary phase. This indicates that the ms^2^ modification may be growth phase dependent and/or that a substrate/cofactor (i.e., sulfur and/or iron) is depleted during stationary phase growth. Additionally, due to the seemingly dynamic nature of the ms^2^ modification as well as its oftentimes low abundance, it is possible that it is not detected during tRNA modification analyses. In *B. subtilis*, the ms^2^t^6^A modification is also more prevalent in exponentially growing cells compared to stationary phase (Vold, 1973; Vold et al., 1982). On the other hand, ms^2^i^6^A exhibits the opposite pattern where ms^2^i^6^A levels are high relative to i^6^A in stationary phase compared to exponential phase in *B. subtilis* (Vold, 1978).

In summary, we confirm that the e-MtaB MTTases in archaea catalyze the synthesis of ms^2^hn^6^A as well as ms^2^t^6^A. Future biochemical and structural work will be required to further understand the substrate specificity determinants of these enzymes as well as combining physiological and genetic experiments to uncover potential regulatory aspects of the ms^2^ tRNA modification.

## Data availability statement

The original contributions presented in the study are included in the article/Supplementary material, further inquiries can be directed to the corresponding author.

## Author contributions

KB: Conceptualization, Investigation, Methodology, Writing – review & editing. T-AD: Investigation, Methodology, Writing – review & editing. KA: Conceptualization, Formal analysis, Funding acquisition, Investigation, Methodology, Project administration, Resources, Supervision, Visualization, Writing – original draft, Writing – review & editing.
