# Correction: Biochemical and genetic studies define the functions of methylthiotransferases in methanogenic and methanotrophic archaea

**DOI:** 10.3389/fmicb.2026.1977300

**Published:** 2026-09-17

**Authors:** Kaleb Boswinkle, Thuc-Anh Dinh, Kylie D. Allen

**Affiliations:** Department of Biochemistry, Virginia Tech, Blacksburg, VA, United States

**Keywords:** methanogens, ANME archaea, methylthiotransferases, radical SAM enzymes, tRNA modifications

In the published article, the locus tag for MTTase was erroneously given as “C4B56_06395”. The correct locus tag is “C4B56_05415”.

A correction has been made to the **Abstract**:

“Here, we report the *in vitro* enzymatic activities of an MTTase (C4B56_05415) from a thermophilic *Ca*. Methanophagales anaerobic methanotroph (ANME) as well as the MTTase from a hyperthermophilic methanogen—MJ0867 from *Methanocaldococcus jannaschii*. Both enzymes catalyze the methylthiolation of *N*^6^-threonylcarbamoyladenosine (t^6^A) and *N*^6^*-*hydroxynorvalylcarbamoyladenosine (hn^6^A) residues to produce 2-methylthio-*N*^6^-threonylcarbamoyladenosine (ms^2^t^6^A) and 2-methylthio-*N*^6^*-*hydroxynorvalylcarbamoyladenosine (ms^2^hn^6^A), respectively.”

A secondary correction has been made to **Results**, Identification and initial characterization of an ANME-1 MTTase, Paragraph 2:

“Analysis of the genome revealed a likely MTTase—C4B56_05415 (PXF52554.1)—that shares 63% identity and 78% similarity to BSM_21210.”

Finally, the Supplementary material file has also been edited to replace any instance of C4B56_06395 to the correct locus tag of C4B56_05415.

The original version of this article has been updated.

