# Supplementary material for: Biochemical and genetic studies define the functions of methylthiotransferases in methanogenic and methanotrophic archaea
Source: Front Microbiol. 2023 Nov 23;14:1304671. doi: 10.3389/fmicb.2023.1304671 (PMC10702137; doi:10.3389/fmicb.2023.1304671)
Supplement: Supplementary file 1 [file Data_Sheet_1.pdf]

Supplementary material for:

Biochemical and genetic studies define the functions of methylthiotransferases in methanogenic and methanotrophic archaea

Kaleb Boswinkle, Thuc-Anh Dinh, and Kylie D. Allen*

Department of Biochemistry, Virginia Tech, Blacksburg, VA

*Corresponding author: kdallen@vt.edu, 540-231-5040

Supplemental Methods:

Generation of protein expression constructs. The putative MTTase from the Black Sea mat ANME-1 metagenome *(1)*, BSM_21210 (UniProt Accession: D1J964; GenBank Accession: CBH38644.1), was first cloned into pJAR50CT – a shuttle vector for protein expression in *Methanococcus maripaludis* (derivative of pMEV1*(2)*) – by obtaining the gene sequence with associated overlaps for Gibson assembly as a gBlock from Integrated DNA Technologies (IDT) (Table S1). The gBlock was assembled with NsiI-digested pJAR50CT using NEBuilder HiFi DNA Assembly Master Mix (New England Biolabs) according to the manufacturer's instructions. A portion of the assembly reaction (2 μl) was then transformed into DH5α Mix and Go cells (Zymo Research) followed by selection on LB agar plates containing ampicillin (50 μg/mL). The sequence verified pJAR50CT_BSM21210 construct was used as a template for subsequent cloning into either pJAR46 for expression of BSM_21210 in *M. maripaludis* with an N-terminal hexahistidine tag or into pET15b for expression of the protein with an N-terminal hexahistidine tag in *E. coli*. The primers were designed with associated overlaps for cloning into NsiI-digested pJAR46 and NdeI-digested pET15b (Table 2). For all plasmid constructs created this work, the VeriFi Polymerase 2x master mix (PCRBIO) was used for PCR following the manufacturer's instructions. The PCR product was purified (Zymo DNA clean and concentrate kit) for subsequent use in the HiFi DNA assembly reaction and transformation into DH5α cells as described above.

The G60 ANME-1 MTTase (C4B56_05415; UniProt Accession: A0A2V3JK99; GenBank Accession: PXF52554) *(3)* was cloned into the *E. coli* expression vector, pET15b, by acquiring the gene sequence with associated overlaps for Gibson assembly as a gBlock from IDT (Table 1). The gBlock was assembled with NdeI-digested pET15b using NEBuilder HiFi DNA Assembly Master Mix followed by transformation into *E. coli* DH5α Mix and Go cells with selection on LB

agar plates containing ampicillin (50 μg/mL). MJ0867 (UniProt Accession: A0A832SY65; NCBI Accession: WP_064496643) – the MTTase from *M. jannaschii* – was first cloned into pET15b by PCR amplification of the gene using primers designed with associated overlaps for Gibson assembly (Table 2) and with genomic DNA from *M. jannaschii* as a template. Since MJ0867 did not express well in our routine *E. coli* expression strain for methanogen proteins (*E. coli*-CodonPlus (DE3)-RIL), this putative MTTase was subsequently cloned into pJAR50CT with a C-terminal twin strep tag (pKB604, see methods for construction of this plasmid below) for expression in *M. maripaludis* (see Table 2 for primers).

The sequences of all constructs were verified by Sanger sequencing by the Genomics Sequencing Center at Virginia Tech and/or by Plasmidsaurus (Lexington, KY).

<u>Construction of pKB604</u>. To replace the C-terminal his-tag of pJAR50CT with a C-terminal twin strep tag, the plasmid backbone was first PCR amplified such that the his-tag was removed. The forward primer was designed to bind downstream of the his-tag and contained a 5' AscI restriction site. The reverse primer was designed to bind upstream of the his-tag and contained a 5' NdeI restriction site. This replaced the NsiI site with an NdeI site. To clone in the twin strep tag, a gBlock was designed containing a thrombin site and the strep tag, as well as regions overlapping with the plasmid backbone. The gBlock and 5,025 bp PCR product were assembled using NEBuilder HiFi DNA Assembly Master Mix followed by transformation into *E. coli* DH5α cells. The correct sequence of the cloning region was verified via Sanger sequencing.

<u>High-resolution LC-MS analysis of hn^6A in *M. acetivorans*</u>. To confirm the identity of hn^6A as opposed to m^6t^6A in *M. acetivorans*, we analyzed the tRNA nucleosides on a Waters Synapt G2-S HDMS interfaced with an Acquity I-Class UPLC system with an Acquity BEH C18 column (2.1 mm x 50 mm; particle size, 1.7 μm; maintained at 35 ºC). Solvent A was water with 0.1% formic

acid, and solvent B was acetonitrile with 0.1% formic acid. The flow rate was 0.2 ml/min, and gradient elution was employed in the following manner (time [min], percent solvent B): (0.01, 1), (5, 20), (7, 95), and (8, 95). Two microliters of sample was injected. The mass spectral data were collected in high-resolution MSe continuum mode (nonselective MS/MS acquisition mode). Parameters were a 2.8-kV capillary voltage, a 125°C source temperature, a 350°C desolvation temperature, a 35-V sampling cone, 50-liter/h cone gas flow, a 500-liter/h desolvation gas flow, and a 6-liter/h nebulizer gas flow. The collision energies for the low-energy scans (function 1) were 4 V and 2 V in the trap region and the transfer region, respectively. Collision energies for the high-energy scans (function 2) were ramped from 25 to 45 V in the trap region and 2 V in the transfer region. Data were analyzed using MassLynx software (Waters).

Table S1. List of gBlocks used in this study.

Name of construct	Description	gBlock sequence
pJAR50CT_BSM21210	The MTTase from the Black Sea Mat ANME-1 genome. cloned into the NsiI site of pJAR50CT using NEB HiFi assembly	ATTTTATAGATAACTAATAGGTG<br>AAATGCATGAATCTAACCGAATT<br>ATCAGAGGGCACGGCAAAAGTTT<br>TTATAGAAACTTTCGGCTGCACC<br>GCAAATACCGGTGACACAATGG<br>AAATGCGAGCGATACTAAGAAA<br>TGCCGGTCATGAGATAGTAGAAG<br>AAAGTGAAGCGGATATTGTTATA<br>GTGAATACATGCACAGTAACAAA<br>AAGAACAGAGTTAAATGTGATA<br>AAGAGACTGAATGAGTTAAAAG<br>AGCGTGGTAAAGCGGTAGTTGTG<br>GCTGGTTGCATGGCGGCGGCACA<br>GCCAGAACTGGTAAGAAGCATTT<br>TAGGTGATGATGTAGCAATGGTG<br>ACGCCCCGAGATATACAAGCTAG<br>AGAAAAGCAAAGGCTTGAGTTC<br>GATGGTGTTATTGCGGTGATTAC<br>AATAGCACAGGGTTGCATAGGTA<br>AATGCACTTATTGTATCGTGAAA<br>CAAGCGAGGGGCAAGCTGAAAA<br>GCTACAAGCCGGAAAAGATATG<br>CGAGGCGGTTAAGAGTGCAGTG<br>GAAAGCGGTGCGAACGAGATAA<br>GGATAACATCGCAGGATTCTAGT<br>GCATACGGTTGGGGCAGCACCGA<br>TATAAAACTACCGGCGTTGTTAG<br>AACAGATAACATCCGTGGAAGG<br>CGATTTTAGGATAAGAGTGGGTA<br>TGATGAATCCATTCACGCTGATG<br>CCTATCCTGGACGAACTTCTTGA<br>GGCGTTCAATACCGAAAAAATCT<br>TCAAATTCTTTCACGTGCCTGTGC<br>AATCGGGCTCTGACCGTGTACTG<br>AGAGAGATGAGAAGGAATTACA<br>AAGTCGCTGATTTTGTTGAGATT<br>GTAACAAACATAAGGGCGCGATT<br>CAGACAGAGTACAATATCCACAG<br>ATTTCATCATTGGTTTTCCAACCG<br>AGACGGAAGAGGATTTCTTCGCA<br>TCACTATATTTATTGGAGGAAAT<br>AAAGCCCGAAAAAGTGAACATA

		ACGCGGTTCTCACCAAGGCCCAG GACAGAAGCATCAAAGTTAACG GACTTGTTGGAACGGAGAAGA AGCGAAGGTCGAGAATATTCTCT ACCGGTTATCACAATATTGTGTT CGCGAAGAACAAGGAATTAGAA GGGGCAGAACTACCTGTTTTAGT AACAGAGCCGGGAAAAAAGGGA GGCGTAATAGCTCGTGACTCAGC GTATAGGGCGATAGTACTAAAAG ATGATTTGCCGCTCGGAGCCCAT TATAACGTAAGGGTAAAGGAGG CGAAGAGCACTTATTTAGTGGCT GATATTTTACAACATTCGTTCAG AACTTCACCATCTGTGCATGCGG CACACCATCCACGAAGATCACAT TAGTGCATGGGGCGCGCCATCGA AGGTCGT
pET15b_G60 ANME-1 MTTase	The MTTase from the G60 thermophilic ANME-1 genome (C4B56_06395, (PXF52554.1) cloned into NdeI-digested pET15b using NEB HiFi assembly	CTGGTGCCGCGCGGCAGCCATATGA GCGAGATTATCGGGAGTTATGGCTC AGGAGCAGAAACAGGAGCAGGAAC AAGAGCAAGAGTCCACATAGAGAC CTTTGGCTGCACTGCAAATGCCGGC GATACGCAGAAACTGCGCGCTATAT TGAAGAGGAGTGGATATCAACTGGT AACGGACTATAAAAAGCCGATTGT GTCATCGTGAACACATGCACAGTGA CGAAGCGGACGGAACTGAATGTGGT GAAGCGGCTGGAGGAACTGAAGAA GCAGGGCAAGCATGTAATCGTAGCC GGCTGTATGGCTGCGGCACAGCCCG AACTGGTGAAGAGCGTTCTGGGGAA GGATACAATTATGATAACGCCTGAA GAGCTATACCCGGTTATGGATTTTG ATTTTGACAACAACTCCTCCGTGGTT GGTATAATCCCGATATCAATGGGCT GTCTCGGAGAATGCACCTATTGCAT TGTGAAGAGAGCGAGGGGGAGACT GAAGAGCCTGAGTCCCGATAGGATA TGCAGCGCGTTAAGGTCCGCAGTGG CGGCTGGTGTGAAGGAAATAAGAAT AACAGCCCAGGATTGTGCCGCTTAC

		GGGTTCGACCGCACGGATATGGGTA TGGATATGGCTGTGAAACTACCTGA GCTCCTGCGGATGCTGACCGAAGTG GAAGGCGATTTTAGAATTCGTATAG GCATGATGAACCCCTTCACACTACT CTGCATCATAGATGAGCTCCTTGAG GCTTTTGAATCCGATAAGGTGTTCA AGTTCTTCCACATACCGGTTCAGTCG GGCTCTGATAAGGTGCTAAGCGATA TGCGCAGGAATTACAAAGTCGCTGA CTTCGTAGAGATAGTGAGAAGAATA AGGAGGCGATTCCATTACTGCACCA TCTGTACAGATTTTATCGTTGGATTT CCAACAGAGGATGAGGATGATTTCC GGGCGTCACTGCAGCTACTGGAGGA AGTGAAGCCTGAGAAGGTGAACATT ACGCGATTCTCACCGCGACCGGGGA CGGAGGCATCGAAACTCAAGGACAT GCTGGCTCGCGATAAGAAGATGAGG TCGCGAACGATGTCGGCGATTTATC ACAGGATGGCACTTGAAGCCAATAA CCAACTGATAGGAGCGGAACTACCG GTTCTGGTTACCGAACGCGGTGATA AAGGCGGTGTGATTGCCCGTGACCC ATCTTATAAGACGATAATATTGGAG GAGGATTTGCCGCCCGGTTCATTCC ATAAGGTGCGTGTGAAGGATGCGAG GAGCACTTATCTGGTTGCCTCTGTGA TTTCATGATGCTCGAGGATCCGGCT GCT
pDN201_MA1153 deletion construct – sgRNA gBlock 1	sgRNA with associated overlaps for assembly into AscI-digested pDN201	CCTTTTGGAGCCTTTTTTTTTCGAAG TTTAAACCTGCAGGCGCGCCAACAA CATCAGTCACCTAAAAAGAGAAAAC GAATTACACGATCACTAATTTTAAA TTTTATATATGTTGACTGAGATTGCA AATTTGAACATTGAAATTTTTTACCC GCTTGATCTGAATAATGACATTGTTC AAAAAAAGTACAAATGATAAAAAA GAAAGCTTCTCAAAAAACAGTAAAG AAGTTCTCCCCAAAATCACCTCAAA AATTCAGAGCTCTATTATCAGAAAA

		AGCGAGCTTAAAAAATTCAAAGGAA AGATACCCCTCTGCACCCTCAAAAT TTTAGACCCTGTGTTGACCTGTAAA AATCAGGAAAAAATTTCCGTCGGTT ATGGTATATGTGATGATTTCCCCTAA TTATGCTGTAAGCACATGTCTGCCTC TCCGGCAGCAGCAGTTTTAGAGCTA GAAATAGCAAGTTAAAATAAGGCTA GTCCGTTATCAACTTGAAAAAGTGG CACCGAGTCGGTGCTTTTAACATCA GT
pDN201_MA1153 deletion construct – sgRNA gBlock 2	sgRNA with associated overlaps for assembly into AscI-digested pDN201	GGTGCTTTTAACATCAGTCACCTAA AAAGAGAAAACGAAGTATTCCCCAG GTTTGAGGTGTTTTAGAGCTAGAAA TAGCAAGTTAAAATAAGGCTAGTCC GTTATCAACTTGAAAAAGTGGCACC GAGTCGGTGCTTTTGCCCTCAGTTCT CTTTTTCTTTTTCTTAAACTTCACGC ACTGCACTTTTGTCCTCACTTTTTTC ATGCCGTCAGATTAACTACTTTTTCT ATCCTTGAAATCAGCGGCTTTTCAG CCCTCATGTACCGGCGATCGCGGCC GCTTAATTAATACGACTCACTATAG
pDN201_MA1153 deletion construct – HDR template	HDR template with associated overlaps for assembly into PmeI-digested pDN201 containing the sgRNA components above	TATTAAAGGCTCCTTTTGGAGCCTTT TTTTTTCGAAGTTTAAACAGGGACG AAGCTCCCATGCTTTATATAGGAGA AGAGGTCGGGACAGAGAACGGAGA TCTCGAGGTTGATATTGCAGTCGAC CCCCTGGAAGGCACAAACCTTACGG CAGACGGTTGCCCGGGCTCGGTTGC AGTTATGGCAATGGCTGAAAGGGGA GGGATCTTCCACGGCCCTGATATCT ATATGGACAAGATTGTTGTAGGGCC CGATGTCGTTCGGTATGAAGAAGAG CATCCTGGCGAAAGGATCGACCTTG ATGCGCCTGTCAGTCATAACCTCGA AATCGTTGCAAAAGCCCTCGGAAGA AAGGTTGAGGAACTTGTAGTCGTGA TCCTTGACCGCCCAAGGCATGCCCA GAAGATAACCGAAATTCGGGAAGCT GGAGCCCGTGTGAGGCTGGTTACCG ATGGAGACCTCATGCCAGGCGTTGC AACTGCGGTCCGAGGTTCAGGCATT

		CACGTGGTCATGGGGGCAGGCGGTT CGGGAGAAGCTGTCCTGACTGCTGC AGCAATCAAGATCCTGGGTGGAAAA ATCCTTGCAAGGCTTGTCCTGCCAA CCGTAGCAAACGGTAAAACCAAGG AAAAGATCGACGAAGAAATAGAGG AAAAGATGCCCAGGCTCGAAGCGAT GGGAATTACCCTTGAGAACATTAAT GATATTCTTGATATTGATAAACTTGT TCCTGGTAAGGATGTCATTTTTTCCG CAACAGCCGTAACTCCGGGCCACTT CCTGCGCGAAGTCAACCTTTTCGGA GGCGGGGATGCCAGAGTACACACG GTTTCTATGGGGGCATCCGGAGCTG TCAGGTTCACGGACAGTATCTATAT CAAGGATAAGCGGGAGACTCCACTC TACCTGTAAATCCTTTTTTCATCTTT TTCATCCATTTTCTTTACCCTTTTTTG AAACACAAATTTCAAAAATTTCGAG TTAAAACATGAAGGTCTATCTTGAG AGTTTTGGCTGTTCTGCAAGCCTGGC ATCAGCCGAAATCATGGCTAATGTA GAAATTACGGGTGCAAAGCCCGGCT ATTTCCTGGGGAGGATTATTGATTG AGGTTGAAAATCTTCGTAAAGGATA GGGGCTGCCGTTGAAGGCAGGAAA ATATCAGCAGAAGCAAGGATGGTTT CAACAAAGGATGGAAAATCTCAACC AAAAAAATCAGCAAATAAAAATGA TCAGTTTTTTTCTTCATTCTGCTTTTT TCCCTGTTTAGATGTTTTATTTTCTTC GTTTTTGATTCCTGCTGGTTTTTAAC CATCAAAATCTTTTCTTTTTATTTTA ATTCTTTCCAATCGAAAGTAAAGTA TTTATGTATATAACTCTGTAATATTT TTTACTTTAAAATCTCAGTTTTTCTA AATCTCAATTCTTCTATCGTTTATTT TTCCGGGGTGAAGCACATAGCTGGG AAAAGATCTACCAAGTGTTCCTGTT TTTTTAAGGATACCCGCGCCTATATT CCTTATTCCATAATCGGGATTTTCAT TGTCCTTGCTGCAACCTTCACCTCTG TTTACCTCCTGAAAATGGACAGTGA GGTTGCCGAAACAATCTATACTACA GAGAAAAGCAATCCCAGGAAGACG GCTATATCCCTTGCGGCATCCGATCT

		TGCTCGCTGCCTTAACTATGCCGGA ATGGAGGCTCTGGAATGGCAGGGAG AACACCCTGTTATCCTGCCTGAGGG CTCTCCGGTTGAAAGGTCCTCTGAA GACAATTTTATGGTAACTCCGCAAA ACCAGAACCTTGAGAAAGGAGACA CTCTTCAAATCTCCATAAACATGCCT TCAGATGTCTGGGGCAAAATCGAAT CCCTCTGGAAGAACAGGGATATTGT CCTGGTCGTAAACGACTCTGCCGGA CGGCAAATCAAACGTGTGAACTATG GGCAGGCTACCGGTTTTCTTCAGAA AGTATCTTTTGAAGAATACCTGGAA GTCCCGGAAAGTGCAGAAGCCGGAT ATGCATCCATAGAACTTTATTACGG AGACGAAAACCTGCAGGCGCGCCA ACAACATCAGTCACCTAAAAAG
pKB604	Twin strep tag with thrombin cleavage site and associated overlaps for assembly into PCR-amplified pJAR50CT	TTATAATTTTATAGATAACTAATAG GTGAAACATATGGGTTCATTAGTTC CAAGAGGATCATGGTCACACCCTCA ATTTGAAAAAGGTGGAGGATCAGGC GGAGGTTCAGGTGGATCAGCATGGT CACACCCACAATTCGAAAAATAAGG CGCGCCTTAAACTTTTAAAAAGTTT AATCAA

Table S2. List of primers used in this study.

Name of construct	Description	Primers (all listed 5' to 3')
pET15b_BSM21210	The MTTase from the Black Sea Mat ANME-1 genome. cloned into NdeI-digested pET15b using NEB HiFi assembly	Fwd: GTGCCGCGCGGCAGCCATATG GAAATGCGAGCG<br><br>Rev: GTTAGCAGCCGGATCCTCGAG CTAATGTGATCTTCGTGGATG
pJAR46_BSM21210	The MTTase from the Black Sea Mat ANME-1 genome. Cloned into *M. maripaludis* expression vector with N-terminal hexahisdine tag using NEB HiFi assembly and the pJAR50CT construct as a template	Fwd: GAATTCACGCGTGAGCTC ATGCATATGAATCTAACCGAA TTATCAGAG<br><br>Rev: GATATCATGAGATCTCTC GAGATG CATCTAATGTGATCT TCGTGGATGG
pET15b_MJ0867	The MTTase from *M. jannaschii*, cloned into NdeI-digested pET15b using NEB HiFi assembly	Fwd: CTGGTGCCGCGCGGCAGCCAT ATGAGAGTATATGTTGAGG<br><br>Rev: AGCAGCCGGATCCTCGAGCAT TAAAGGATAAGCTCCC
pKB604	Replacing the C-terminal his-tag of pJAR50CT with a C-terminal twin strep tag	Fwd: GGCGCGCCTTAAACTTTTAAA AAGTTTAATCAAAACC<br><br>Rev: CATATGTTTCACCTATTAGTT ATCTATAAAATTATAA
pKB604_MJ0867 (pJAR50CTstrep_MJ0867)	The MTTase from *M. jannaschii*, cloned into a *M. maripaludis* expression vector with a C-terminal twin strep tag using using NEB HiFi assembly	Fwd: GATAACTAATAGGTGAAACA TATGAGAGTATATGTTGAGGG CT<br><br>Rev: CTCTTGGAACTAATGAACCCA TAAGGATAAGCTCCCCTTTCA
M. acetivorans ΔMA1153	Primers designed to amplify 1,094 bp upstream of MA1153 and 1,121 bp downstream of MA1153	Fwd: GCTCATTCATGTGACCGAAGC TGCTGCAAT<br><br>Rev:

		CCCGTTAACCTTCCTGACCTT TATCTCTTC

Table S3. Summary of putative MTTase-encoding genes in some ANME-1 vs. ANME-2 genomes. MJ0867 (the MtaB MTTase from *M. jannaschii*) was used as the BLASTp query. The same putative MTTase was identified in all genomes if other MTTases, such as bacterial MtaB, MiaB or RimO, were used as queries.

Genome	Reference	NCBI genome accession number	Putative MTTase gene GenBank accession number (E-value)
**ANME-1**			
ANME-1 Meyerdierks (ANME-1b)	Meyerdierks et al. 2010 *(1)*	FP565147.1	CBH38644/BSM_21210 (2e-87)
ANME-1 G37 (ANME-1a)	Krukenberg et al. 2018 *(3)*	GCA_003194425.1	PXF51001.1 (2.5 e-73)/ PXF51817.1 (8.6e-6)
ANME-1 G60 (ANME-1a)	Krukenberg et al. 2018 *(3)*	GCA_003194435.1	PXF52554.1(5e-87)
NA091.008_bin2_ ANME-1 (ANME-1c)	Laso-Perez et al. 2023 *(4)*	GCA_026134085.1	MCW7078476 (3e-82)
**ANME-2**			
ANME-2a	Wang et al. 2014 *(5)*	IMG genome ID: 2566123520	MBC2697835.1 (1e-77)
ANME-2b CONS3730F09p3b1	Chadwick et al. 2022 *(6)*	OCYX01000000	KAF5423722.1 (8-10)/ KAF5421500.1 (2.5e-6)
ANME-2c ERB4	Chadwick et al. 2022 *(6)*	MT631448	QNO48029 (1e-8)
Ca. Methanoperedens nitroreducens (ANME-2d)	Haroon et al. (2013) *(7)*	GCF_000685155.1	KCZ71469.1 (1e-8)/ KCZ71973.1 (7.6e-4)

Table S4. Analysis of the potential radical SAM sulfur-insertion enzymes found in the G60 ANME-1 genome *(3)*. Only one protein had significant homology to any of the enzymes, and this protein only had homology to the known MTTases.

Protein	pBLAST Hits	E-value
**e-MtaB (MJ0867)**	PXF52554.1	5e-87
**MiaB (*E. coli* K12)**	PXF52554.1	1.5 e-43
**MtaB (*B. subtilis)***	PXF52554.1	6.6e-43
**RimO (*E. coli* K12)**	PXF52554.1	1.9 e-38
**BioB (*E. coli* K12)**	None	N/A
**LipA (*E. coli* K12)**	None	N/A
**LipS1 (TK2109 from *T. kodakarensis*)**	None	N/A

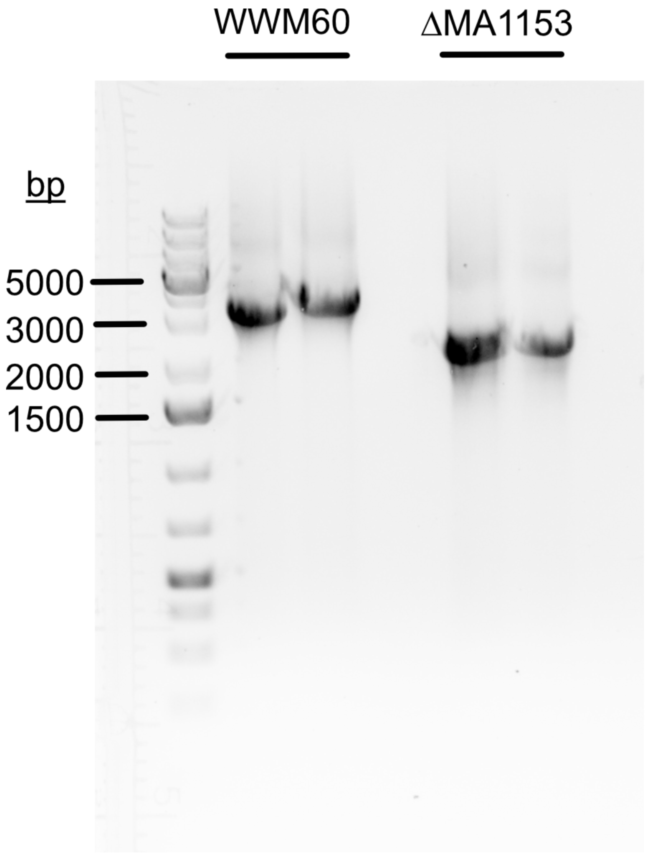

Figure S1. PCR confirmation of the MA1153 deletion in *M. acetivorans* WWM60. Primers were designed to amplify beginning 1,094 base pairs upstream and 1,121 base pairs downstream of the MA1153 gene (Table S2). The expected size of the PCR product for the wild-type (WWM60) is 3,710 bp and the expected size for the deletion strain is 2,215 bp. 60 bp at the 5' end and 60 bp at the 3' end of the gene were left intact in the design of the deletion strain. The PCR product of the deletion strain compared to wild-type was also verified by Sanger sequencing.

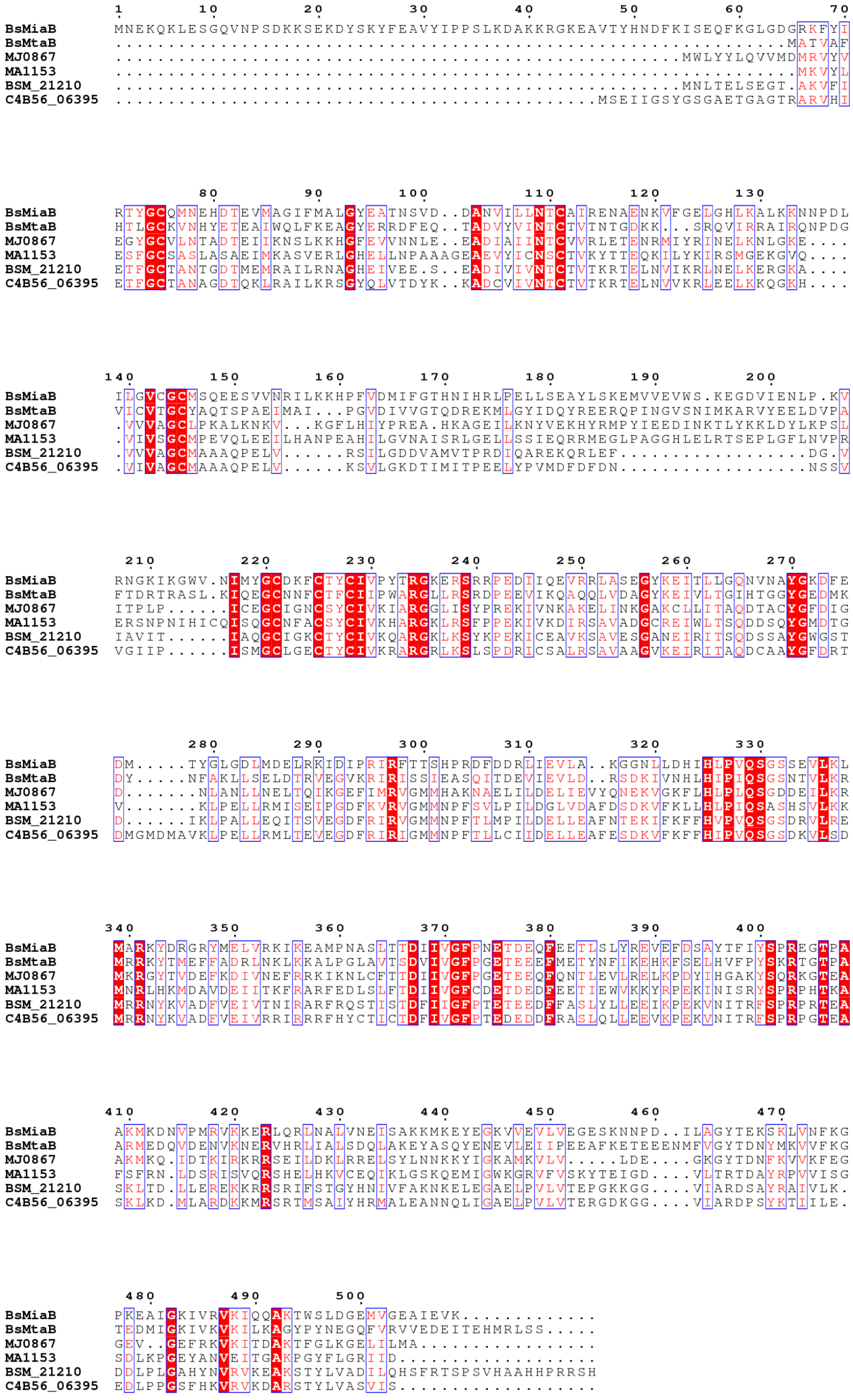

Figure S2. Sequence alignment of selected MTTases. BsMiaB - WP_049140040.1; BsMtaB - QGU23964.1; MJ0867 - Q58277.1; MA1153 - AAM04574.1; BSM_21210 - CBH38644.1; C4B56_06395 - PXF52554.1.

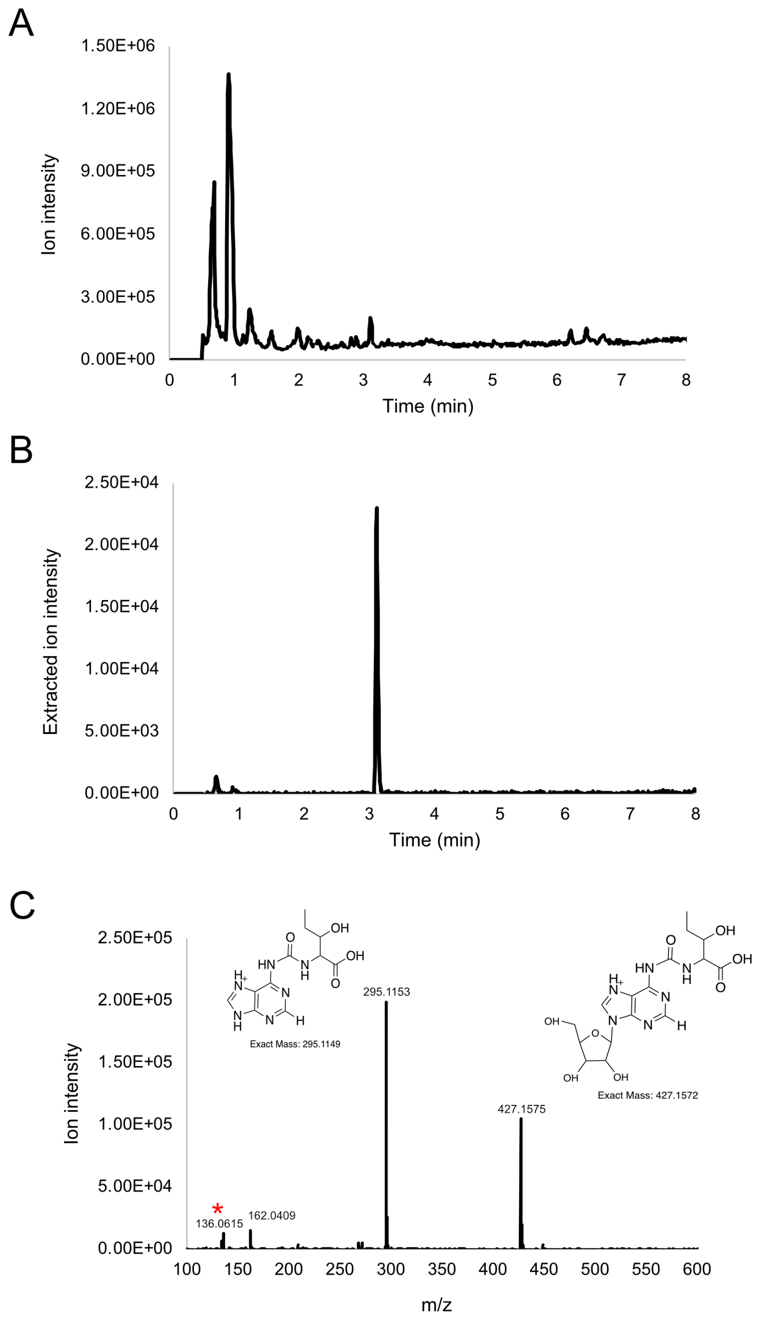

Figure S3. High-resolution LC-MS analysis of hn^{6}A in *Methanosarcina acetivorans*. (A) Total ion current of HR-LC-MS analysis of nucleosides derived from *M. acetivorans* tRNA. (B) Extracted ion chromatogram for *m/z* = 427, corresponding to hn^{6}A. (C) Mass spectrum of *m/z* 427 peak. The fragment ion at *m/z* 136.0615 (red asterisk), corresponding to the protonated adenine nucleobase, confirms the identity of hn^{6}A as opposed to m^{2}t6^A, the latter of which would have a fragment ion corresponding to a methylated adenine (*m/z* 150.077). It is important to note that this high-resolution data was obtained on a different instrument with a different column and LC program compared to other data presented in the main manuscript, so the retention times differ.

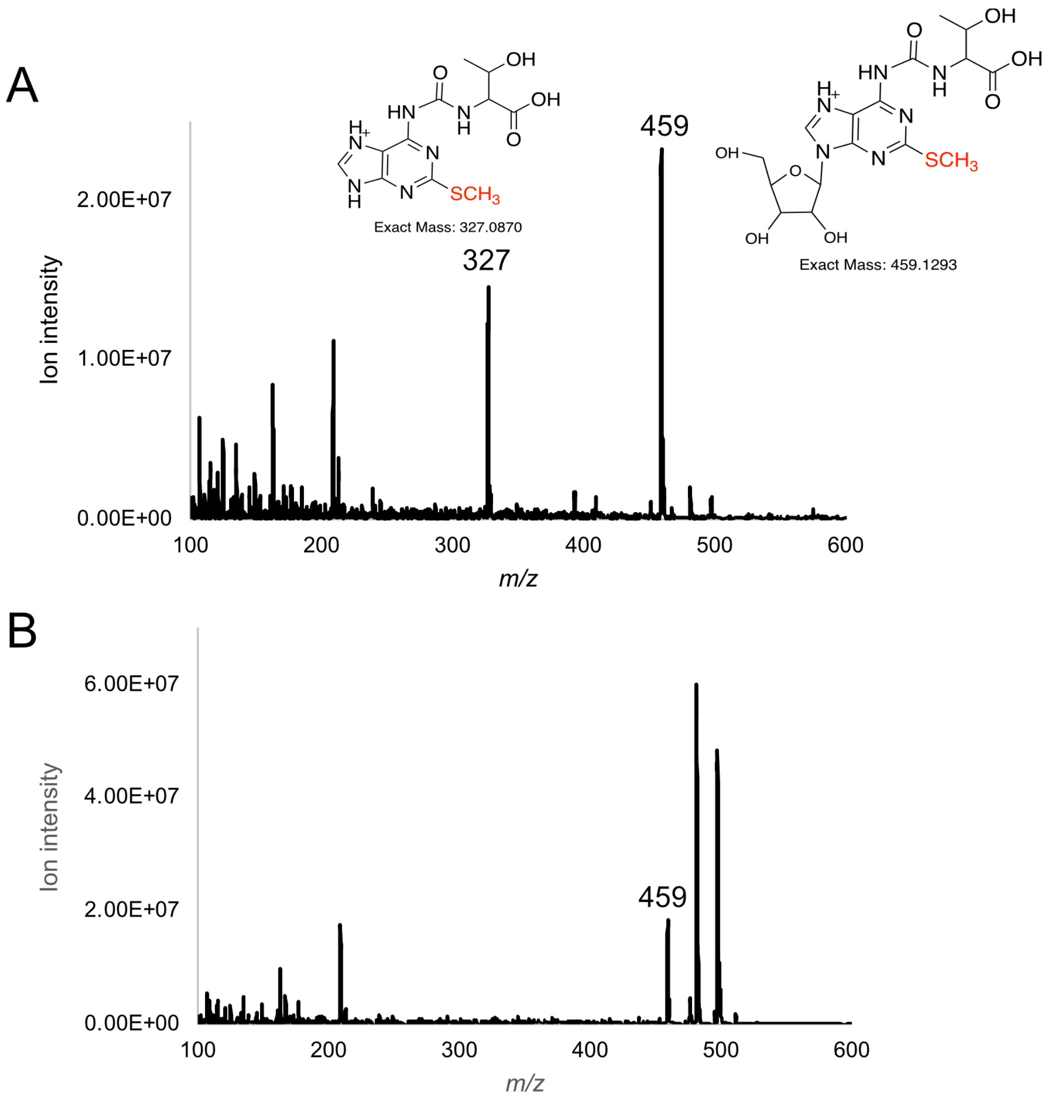

Figure S4. (A) Mass spectra of ms^2t^6A produced in the G60 ANME-1 MTTase *in vitro* enzyme reaction compared to (B) the 459 ion observed in digested tRNA from *M. acetivorans*. The latter spectrum lacks the characteristic base fragment ion at *m/z* 327 and, thus, is not ms^2t^6A. Additionally, as mentioned in the main manuscript, this molecule elutes about 0.8 minutes after ms^2t^6A. This ion is observed in all tRNA preps reported in this work.

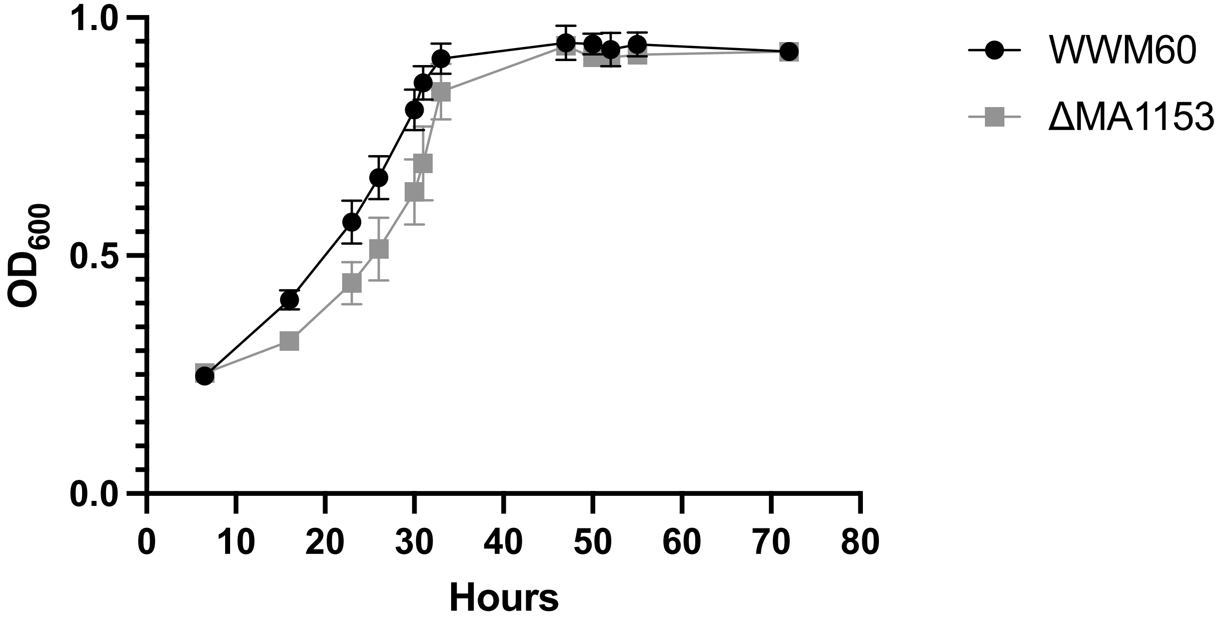

Figure S5. Growth curve of wild-type (WWM60) compared to ΔMA1153 *M. acetivorans* strains grown in triplicate in high salt medium with 100 mM methanol at 37 °C. It is important to note that the gene editing plasmid was not cured from the ΔMA1153 strain investigated in this study, but it was grown here in the absence of antibiotic.

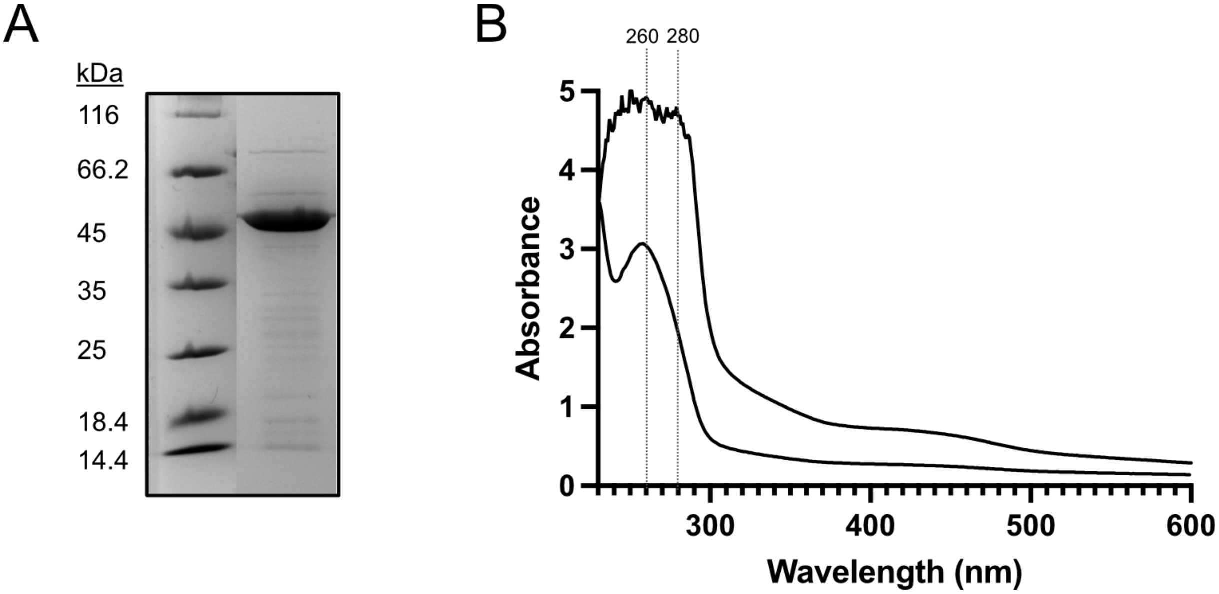

Figure S6. Purification and UV-Vis analysis of *Mj*MTTase. (A) SDS-PAGE gel showing *Mj*MTTase (MJ0867) with a C-terminal twin strep tag expressed and purified from *M. maripaludis*. (B) UV-Vis spectra of purified *Mj*MTTase showing the 420 nm absorbance shoulder due to the presence of [4Fe-4S] clusters as well as a significant 260 nm peak due to the presence of RNA bound the purified protein (as confirmed by LC-MS in Fig. S7).

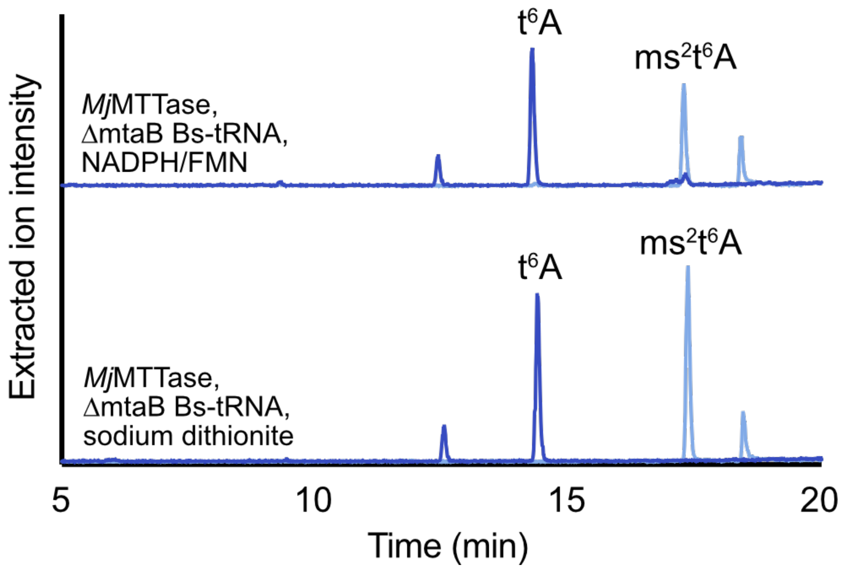

Figure S7. *In vitro* enzymatic activity of *Mj*MTTase with bulk tRNA from *B. subtilis* Δ*mtaB*. Top spectrum shows the activity using the NADPH/FMN reducing system *(8)* and the bottom spectrum shows the activity using the traditional strong chemical reductant, sodium dithionite.

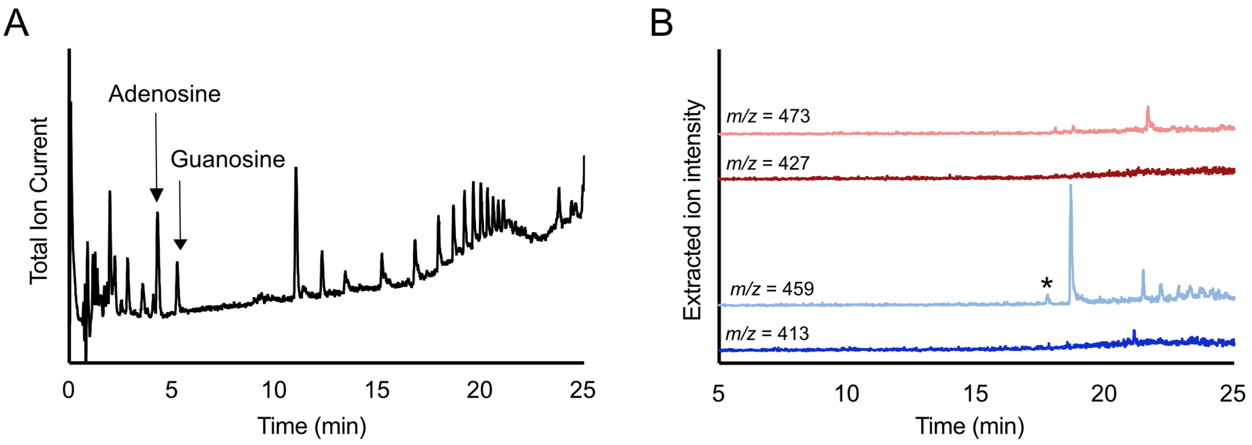

Figure S8. *Mj*MTTase control reaction with all reaction components except added tRNA. (A) Total ion current chromatogram with peaks for adenosine and guanosine highlighted, confirming that the purified enzyme contains bound RNA. (B) Extracted ion chromatograms demonstrating the absence of modified nucleosides of interest. A small peak exists that may be ms^2t^6A (asterisk), but it is in too low abundance to confirm. Overall, this control experiment shows that the activity reported in Figure S7 above and Figure 7 of the main manuscript is a result of the added tRNA and not from background RNA bound to the purified enzyme.

References

([1.) Meyerdierks, A., Kube, M., Kostadinov, I., Teeling, H., Glockner, F. O., Reinhardt, R., and Amann, R. (**2010**) Metagenome and mRNA expression analyses of anaerobic methanotrophic archaea of the ANME-1 group, *Environ. Microbiol. 12*, 422-439.

([2.) Sarmiento, F., Leigh, J. A., and Whitman, W. B. (**2011**) Genetic systems for hydrogenotrophic methanogens, *Methods Enzymol. 494*, 43-73.

([3.) Krukenberg, V., Riedel, D., Gruber-Vodicka, H. R., Buttigieg, P. L., Tegetmeyer, H. E., Boetius, A., and Wegener, G. (**2018**) Gene expression and ultrastructure of meso- and thermophilic methanotrophic consortia, *Environ. Microbiol. 20*, 1651-1666.

([4.) Laso-Perez, R., Wu, F., Cremiere, A., Speth, D. R., Magyar, J. S., Zhao, K., Krupovic, M., and Orphan, V. J. (**2023**) Evolutionary diversification of methanotrophic ANME-1 archaea and their expansive virome, *Nat Microbiol 8*, 231-245.

([5.) Wang, F. P., Zhang, Y., Chen, Y., He, Y., Qi, J., Hinrichs, K. U., Zhang, X. X., Xiao, X., and Boon, N. (**2014**) Methanotrophic archaea possessing diverging methane-oxidizing and electron-transporting pathways, *The ISME journal 8*, 1069-1078.

([6.) Chadwick, G. L., Skennerton, C. T., Laso-Perez, R., Leu, A. O., Speth, D. R., Yu, H., Morgan-Lang, C., Hatzenpichler, R., Goudeau, D., Malmstrom, R., Brazelton, W. J., Woyke, T., Hallam, S. J., Tyson, G. W., Wegener, G., Boetius, A., and Orphan, V. J. (**2022**) Comparative genomics reveals electron transfer and syntrophic mechanisms differentiating methanotrophic and methanogenic archaea, *PLoS Biol. 20*, e3001508.

([7.) Haroon, M. F., Hu, S., Shi, Y., Imelfort, M., Keller, J., Hugenholtz, P., Yuan, Z., and Tyson, G. W. (**2013**) Anaerobic oxidation of methane coupled to nitrate reduction in a novel archaeal lineage, *Nature 500*, 567-570.

([8.) Eastman, K. A. S., Jochimsen, A., and Bandarian, V. (**2023**) Intermolecular electron transfer in radical SAM enzymes as a new paradigm for reductive activation, *J. Biol. Chem.*, 105058.
